# Canonical Wnt Signaling Promotes Early Hematopoietic Progenitor Formation and Erythroid Specification during Embryonic Stem Cell Differentiation

**DOI:** 10.1371/journal.pone.0081030

**Published:** 2013-11-26

**Authors:** Anuradha Tarafdar, Edwina Dobbin, Pamela Corrigan, Robin Freeburn, Helen Wheadon

**Affiliations:** 1 Paul O’Gorman Leukaemia Research Centre, University of Glasgow, United Kingdom; 2 Biomedical Science Institute, University of Ulster, Northern Ireland, United Kingdom; 3 Department of Haematology, Western General Hospital, Edinburgh, United Kingdom; 4 School of Science, University of the West of Scotland, Paisley, United Kingdom; University of Houston, United States of America

## Abstract

The generation of hematopoietic stem cells (HSCs) during development is a complex process linked to morphogenic signals. Understanding this process is important for regenerative medicine applications that require *in vitro* production of HSC. In this study we investigated the effects of canonical Wnt/β-catenin signaling during early embryonic differentiation and hematopoietic specification using an embryonic stem cell system. Our data clearly demonstrates that following early differentiation induction, canonical Wnt signaling induces a strong mesodermal program whilst maintaining a degree of stemness potential. This involved a complex interplay between β-catenin/TCF/LEF/Brachyury/Nanog. β-catenin mediated up-regulation of TCF/LEF resulted in enhanced brachyury levels, which in-turn lead to Nanog up-regulation. During differentiation, active canonical Wnt signaling also up-regulated key transcription factors and cell specific markers essential for hematopoietic specification, in particular genes involved in establishing primitive erythropoiesis. This led to a significant increase in primitive erythroid colony formation. β-catenin signaling also augmented early hematopoietic and multipotent progenitor (MPP) formation. Following culture in a MPP specific cytokine cocktail, activation of β-catenin suppressed differentiation of the early hematopoietic progenitor population, with cells displaying a higher replating capacity and a propensity to form megakaryocytic erythroid progenitors. This bias towards erythroid lineage commitment was also observed when hematopoietic progenitors were directed to undergo myeloid colony formation. Overall this study underscores the importance of canonical Wnt/β-catenin signaling in mesodermal specification, primitive erythropoiesis and early hematopietic progenitor formation during hematopoietic induction.

## Introduction

Wnt proteins are highly insoluble glycoproteins, which remain associated with the cell surface or extracellular matrix of the cell following secretion [1]. Consequently, Wnt proteins tend to act in an autocrine or spatially confined paracrine fashion, targeting cells in close proximity. The expression of Wnt target genes is regulated by nuclear β-catenin that is bound to the transcription factors of the TCF/LEF family [2]. Canonical Wnt/β-catenin signaling is essential for orientating the anteroposterior axis and generating mesoderm which precedes primitive hematopoiesis [3,4]. Wnts, their receptors and active β-catenin are highly expressed in embryonic hematopoietic tissues indicating an essential role for Wnt signaling in developmental hematopoiesis [1]. Recent studies have clarified the role of individual Wnts during hematopoietic ontogeny and more importantly how canonical Wnt signaling affects primitive and definitive hematopoietic specification. Wnt 16 has been demonstrated to play a role in the earliest specification of hematopoietic stem cells (HSCs) [5] which correlate to the high expression observed in the Aorta, Gonads and Mesonephros region (AGM) during mouse development [6]. Wnt 5a has also been identified as a regulator of early hematopoietic stem cells and is highly expressed in early mouse embryonic tissues (Yolk Sac (YS) and AGM), and primitive hematopoietic progenitors [1,7,8]. In *Xenopus* Wnt 4 has been identified as essential for the formation and maintenance of the ventral blood islands [9]. Interestingly Wnt 3a is tightly regulated only being detected in the AGM and for a defined period E15-16 in the fetal liver (FL) [7,8]. This corresponds to the stages when the first HSCs are generated and then migrate and undergo a large expansion in the FL [10,11]. Wnt 3a deficiency results is embryonic lethality [12] with analysis of FL at E12.5 of embryonic development revealing that this is accompanied by reduced numbers of long-term HSC and multipotent progenitors (MPP), which are severely and irreversibly impaired in long-term reconstitution capacity as observed in serial transplantation assays [13]. Canonical Wnt signaling has recently been shown to be essential for HSC generation in the AGM at E10.5, with hematopoietic precursors arising from CD31^+^, c-Kit^-^ endothelial-like precursors that express VE-cadherin. Subsequent to this stage β-catenin signaling is dispensable for HSC establishment but can modulate HSC behavior as demonstrated by increased reconstitution potential following activation of the pathway [14]. These studies support a key role for canonical Wnt pathway in the early stage of establishing hematopoiesis in vertebrates.

In adult hematopoiesis, loss- and gain- of function of β-catenin combined with cre-mediated recombination to target the HSC population has produced conflicting views regarding the importance of β-catenin for normal HSC function. Conditional deletion of β-catenin using Vav-1-Cre impaired HSC self-renewal [15,16], supporting studies using the Wnt-negative regulator Dickkopt 1 [17] and Wnt3a deficient mice [13]. Whereas Mx-Cre-mediated deletion of β-catenin [18] or β- and γ-catenin simultaneously had no effect on hematopoiesis [19,20]. Similarly gain of function approaches have been controversial with stabilized forms of β-catenin either resulting in exhaustion of the HSC pool and failure to reconstitute the hematopoietic system in transplantation assays [21,22] or enhancement of HSC function and maintenance of an immature phenotype [15,23-25].These findings may be explained in part by the differing levels of canonical Wnt signaling achieved using these systems. Indeed combinations of hypomorphic allele mutations and a conditional deletion allele of the adenomatous polyposis coli (APC) gene to modulate *in vivo* canonical Wnt signalling, revealed that mild to moderate activation of the pathway is advantageous resulting in increased clonogenic and differentiation potential with greater reconstitution potential of HSCs, whereas high levels of activation resulted in a differentiation block and failure to reconstitute irradiated recipient mice [26]. 

Although these studies provide important information on the role of canonical Wnt/β-catenin signaling in steady state homeostasis the ability to study early embryonic hematopoiesis has been hindered by the inability to specifically over-express/delete key components of this pathway during embryogenesis. Mouse embryonic stem (ES) cells therefore provide an ideal *in vitro* model for studying both the initiation of primitive hematopoiesis and later stages when more definitive hematopoiesis becomes established [8,27-29]. Following the induction of differentiation, ES cells generate colonies known as embryoid bodies (EB) containing developing cell populations of all three germ layers [29-31]. Mesoderm-derived populations within these developing EB can be directed to form hemangioblasts [32–34] with the capacity to undergo further hematopoietic lineage commitment to form myeloid, erythroid and lymphoid cells. This system has been well characterized through gene expression and progenitor cell analysis and shown to closely parallel hematopoietic commitment during embryogenesis [8,33,34]. Using ES differentiation models, it has previously been demonstrated that Wnt, BMP and Activin signaling are important for establishing primitive hematopoietic commitment via the Cdx-Hox axis with Wnt signaling being involved in primitive erythroid colony formation [9,35-37].

To characterise the role of the canonical Wnt/β-catenin signal transduction pathway in early cell specification and more specifically early hematopoietic differentiation, we have utilised ES cells as an *in vitro* model. Activation of the pathway at different stages of differentiation was achieved using complementary pharmacological and genetic approaches. We demonstrate that β-catenin dependent signaling induces a strong mesodermal program whilst maintaining a degree of stemness potential during early differentiation induction. This is accompanied by a strong induction of genes involved in primitive hematopoietic development. When directed to undergo hematopoietic differentiation, β-catenin signaling enhanced this process by promoting early hematopoietic and MPP, megakaryocytic erythroid progenitors (MEP) and erythroid colony formation. Overall, we demonstrate that the canonical Wnt pathway enhances developmental hematopoiesis processes, especially primitive and more definitive erythropoiesis.

## Materials and Methods

### Cell culture and generation of transfectants

Dominant positive ΔGSKβ-catenin (DP-βC), with the CK1 and GSK-3 binding sites Serine 33, 37, 45 and threonine 41 mutated to alanine by site-directed mutagenesis, (Kindly provided by Dr. Barth, Stanford, USA) was cloned into pUHD10-3 neomycin and transfected into E14tga murine ES cells expressing the tetracycline-sensitive transactivator, tTA. The β-catenin mutation resulted in a dominant positive form, (DP-βC), resistant to proteosomal degradation. Culture, selection and screening of clones were performed as previously described [38]. For the induction of DP-βC, cells were washed x3 in PBS and incubated in the absence of tetracycline for 24 h or as indicated.

### Proliferation & self-renewal assays

XTT bioreduction assays and trypan blue exclusion were performed as previously described [38] to assess the IC_50_ of the pharmacological inhibitors 6-bromoindirubin-3’oxime/BIO, and XAV939 (Calbiochem). Self-renewal of parental ES cells plus the pharmacological inhibitor 5 μM BIO, 5 μM & 10 μM XAV, or dimethyl sulfoxide alone and DP-βC ES cells plus and minus tetracycline were analyzed using alkaline phosphatase staining. Cells were washed, fixed in methanol and then stained for 15 minutes with 1 mg/mL Fast Red TR salt TM (Sigma) dissolved in 0.1 M Tris pH 9.2 containing 200 µg/mL Napthol AS-MX phosphate. 

### RT-PCR and TaqMan Mouse Stem Cell Pluripotency Array cards

Total RNA was prepared using RNAeasy Plus extraction kit (Qiagen). RNA (1 μg) was reverse-transcribed using Superscript reverse transcriptase and oligo dT primers (Invitrogen Life Technologies). Semi-quantitative PCR was performed using 2 μL cDNA and standard conditions using gene-specific primers with non-saturating cycle-numbers (24-32 cycles). Quantitative PCR was performed using 2 μL cDNA with gene specific primers (Table S1) and 2x TaqMan Universal PCR Master Mix (Applied Biosystems) on an Applied Biosystems Prism 7900HT system. The 2^-ΔΔCT^ method was used to calculate relative expression levels for each gene. RNA was reverse-transcribed using a High Capacity cDNA Reverse Transcription kit (Applied Biosystems) and PCR performed using the Applied Biosystems® TaqMan® Mouse Stem Cell Pluripotency Array (4385363) as per manufacturer instructions. Data was quantified using RQ Manager Analysis software. Relative gene expression was calculated using the 2^-ΔΔCT^ method. Genes included for analysis had a CT value ranging between 18-35, with the ΔCT calculated using the average CT from five endogenous controls as reference genes. Following calibration using the control samples (-DP-βC or -BIO) the RQ ratio (arbitory units) of the test sample (+DP-βC or +BIO) were plotted as fold increase/decrease as a measure of mRNA gene expression. Transcriptional changes of >2.0 fold change were included in the analysis.

### Immunoblotting and antibodies

Immunoblotting was performed for; Phospho-STAT 3 (9131), Phospho-Akt substrate (9614), GAPDH (2118), TCF1, TCF4 & LEF1 (9383) (Cell Signaling Technologies), STAT 3 (sc-7179), Nanog (sc-134218, Santa Cruz), Total GSK 3 (05-412), Active β-catenin (05-665, Upstate-Millipore), Total β-catenin (610154), anti-GSK3β Tyr 216 (612313, BD Transductions) and Brachyury (20680, Abcam) as previously described [38].

### Flow cytometry

For immunostaining analysis, cells were harvested, resuspended at 0.5 x 10^7^ cells per mL of FACS Buffer (PBS with 2% FCS and 0.02% sodium azide) and incubation with 1 µg of Rat Anti-Mouse CD16/CD32 Fc Block™ (Becton Dickinson, BD 553142) for 30 min, 4°C. For intracellular staining, the cells were harvested, blocked, washed x 2 with FACS Buffer prior to fixing and permeabilization using FIX & PERM^®^ Kit (BD) as per manufacturer’s instructions. Labelling was performed using 0.5 μg of each flurochrome conjugated antibody or relevant isotype control for 1 h, 4°C in the dark. Secondary staining was performed for a further 30 min for any indirect stains. Brachyury (Abcam 20680), Nanog (560277), Oct3/4 (9006287), Sox2 (9006407), CD11b (552850), Flk1 (555308), CD24 (55313137), CD41 (556437), CD44 (560451), CD45 (557235), c-Kit (553356), Sca1 (558162), Gr1 (553129) and Flt3 (553842) all from BD, CD71 (113802), Ter119 (116205, Bio-legend), FITC Secondary (BD 554020), Step-Avidin Secondary (BD 554064) were used. Following washing, cells were analyzed on a FACSCanto™ II flow cytometer (BD) and data analysed using FlowJo software (Treestar). For isolation of hematopoietic populations cells were immunostained and isolated on a FACSAria™ cell sorter flow cytometer (BD).

### Chromatin Immunoprecipitation (ChIP)

ChIP was performed using a previously described method [39]. In brief parental cells +/- BIO or DP-βC cells +/- Tet were cultered for 4 days minus LIF. 20 x 10^6^ cells were formaldehyde treated to cross-link DNA-protein interactions and reaction stopped using Glycine. Cells were lysed in cell lysis buffer and nuclei were pelleted, resuspended in nuclei lysis buffer (NLB) and incubated for 10 min, 4°C. IP dilution buffer (IPDB) was added and nuclei sonication on ice to obtain 300-1000 bp fragments of sheared DNA. Fragments were collected by centrifugation, resuspended in NLB:IPDB and chromatin precleared using 100 µl Rabbit serum, 1 h at 4°C. Protein G-agarose (100 μl of the bed volume) was added and incubated overnight at 4°C. Antibody-agarose complexes were pelleted and the chromatin supernatant divided into five portions, one was stored as input (positive control) and the other four immunoprecipitated with 2 µg; Stat 3, Brachyury, TCF/LEF-1 or IgG (as a negative control) antibody at 4°C overnight. Protein G-agarose (50 μl of the bed volume) was added and incubated for 3 h at 4°C. The Protien G-agarose-complexes were collected by centrifugation, washed x 2 in low salt IP wash Buffer x 2 in high salt IP wash buffer and x 2 with TE buffer, pH 8.0. To reverse the cross links, IP Elution buffer was added. The samples were incubated with RNase A at 65°C for 6 h and proteinase K treated overnight at 45°C. DNA was extracted using phenol chloroform and analysis performed using PCR with ChIP specific primers (Table S2 *(i)* & *(ii)*).

### Hematopoietic differentiation

Primary EB (Day 4) and early hematopoietic progenitor formation (Day 8) were carried out as previously described [8]. For primitive erythroid colony formation EB’s were harvested [40,41] and a single cell suspension plated at 2.5 x 10^4^/mL in methylcellulose medium containing IL-3, IL-6, EPO and SCF (M3434; StemCell Technologies). Colonies were scored following 7 days in culture. For MPP colony formation [42], early hematopoietic progenitors (Day 8) were harvested and a single cell suspension plated at 2.5 × 10^4^/mL in methylcult supplemented with 1% BSA fraction V and cytokines to promote myeloid progenitor colony formation; 25 ng/mL GM-CSF, 100 ng/mL SCF, 10 ng/mL IL-3, 10 ng/mL IL-11, 10 ng/mL TPO, 10 ng/mL Flt3 (PeproTech™), and 1 U/mL EPO (R&D Systems Ltd.) for 7 days. The colonies were replated in the same cocktail for a further 7 days to assess replating ability. To promote granulocyte/macrophage differentiation, early hematopoietic progenitors (Day 8) were replated at 2.5 × 10^4^/mL in methylcult with 1% BSA and cytokines to promote both myeloid and erythroid colony formation; 25 ng/mL GM-CSF, 25 ng/mL G-CSF, 10 ng/mL SCF, 10 ng/mL IL-3 and 2 U/mL EPO for 7 days. Hematopoietic colonies were scored using a Nikon Eclipse TS100 microscope and digital camera system. Cells were harvested at day 0 (ES), day 4 (early differentiation induction) day 8 (early hematopoietic progenitor) and day 15 (MPP, myeloid colonies) for RNA extraction and FACS analysis.

## Results

### Canonical Wnt/β-catenin signaling influences early cell fate decisions

**Figure 1:**
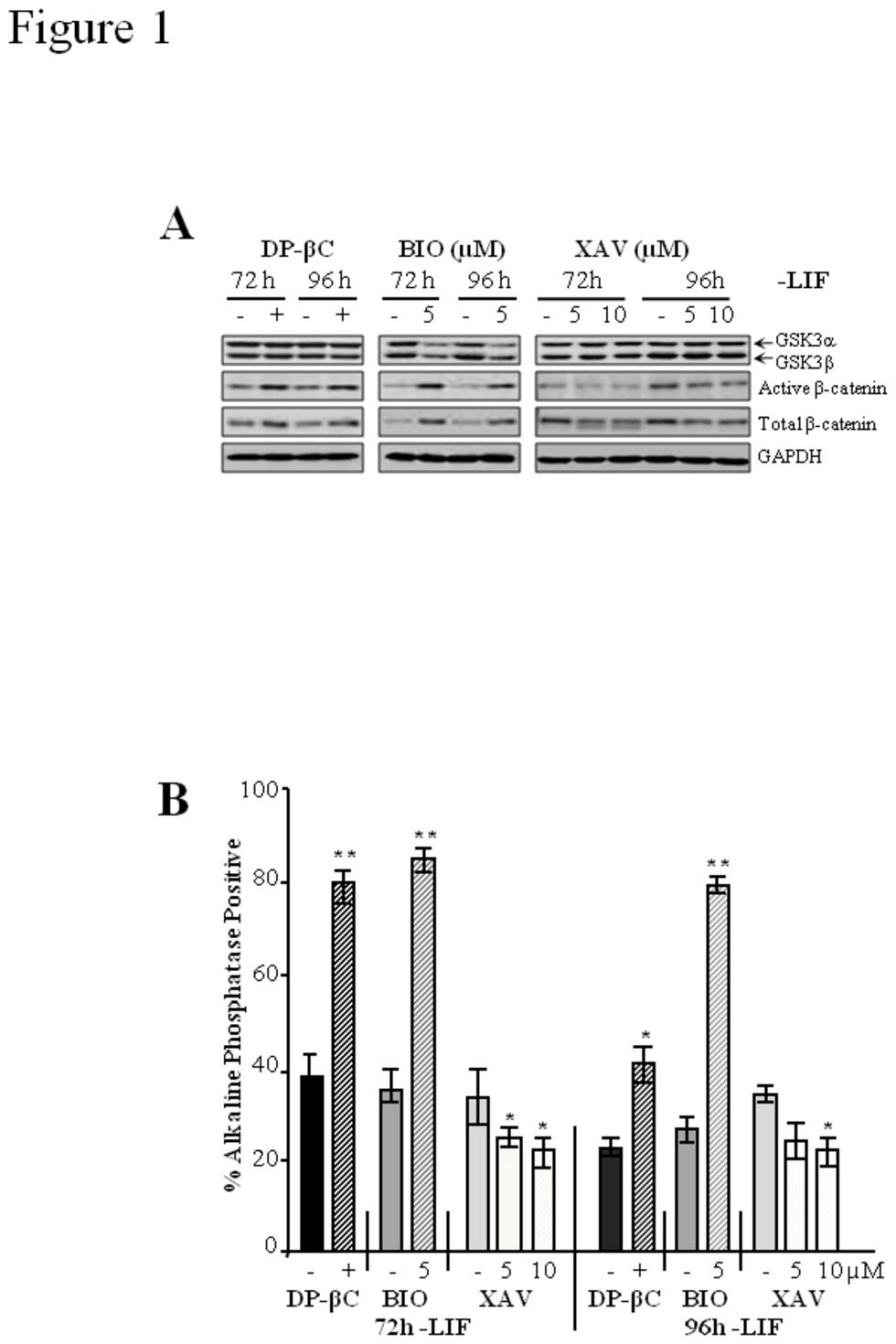
Activation of the canonical Wnt pathway maintains self-renewal in the absence of LIF. Parental E14 ES cells were cultured with or without BIO (5 µM) or XAV (5 or 10 µM) and DP-βC ES cells with or without Tet for 72 and 96 hours in the absence of LIF. (A) Protein extracts were immunoblotted for key signaling proteins involved in the Wnt pathway regulation; GSK-3, Active and total β-catenin with GAPDH used as a loading control, (Representative gel images shown, n=3). (B) Self-renewal potential was assessed by the percentage of colonies staining positive for alkaline phosphatase (AP) is given (Mean ^+^ SEMs, n=3 * p<0.05, ** p<0.005 by paired students t-test).

**Figure 2:**
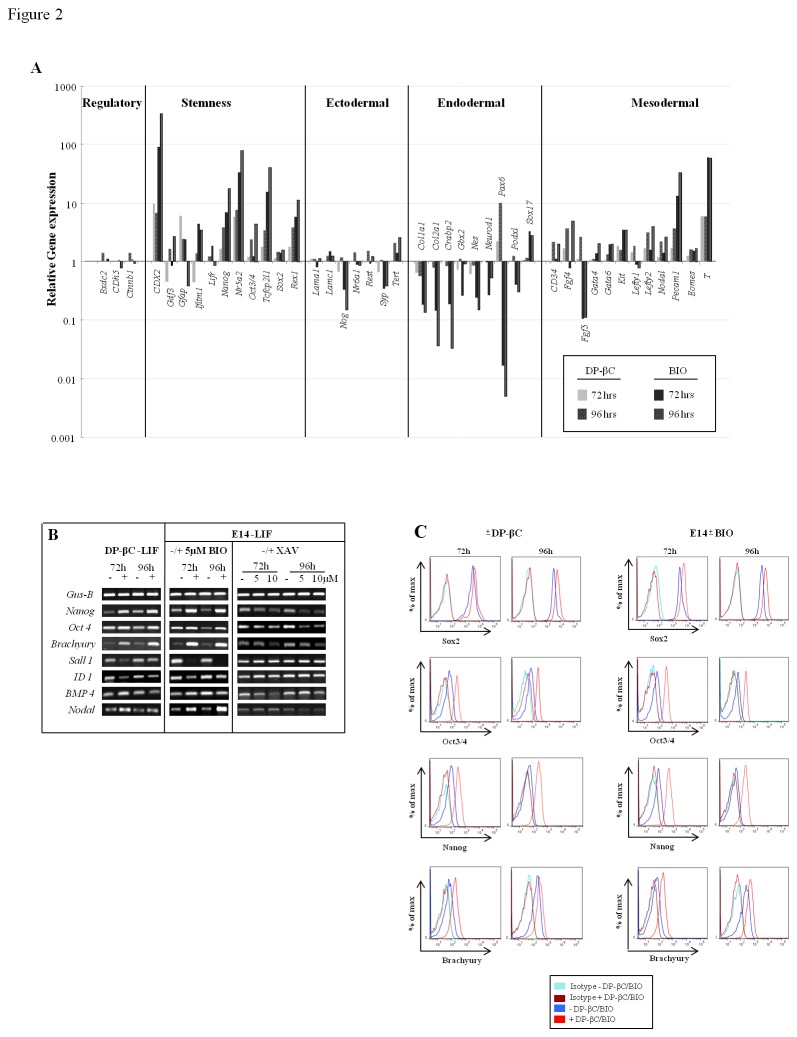
Active β-catenin directs mesodermal differentiation. (A) Relative gene expression profile TaqMan® Mouse Stem Cell Pluripotency Array of mRNA harvested from DP-βC (+/- Tet) or E14 ES cells (+/- 5 µM BIO) following 72 and 96 hours without LIF. Relative expression of each gene calibrated to untreated controls (-DP-βC or -BIO) using the 2 ^–ΔΔCT^ method, plotted on a log scale with a relative expression of 1 representing no change to gene expression (Mean ^+^ SEMs, n=3). (B) Semi-quantitative RT-PCR for DP-βC (+/- Tet) and E14 ES cells (+/- 5 µM BIO, +/- 5 µM & 10 µM XAV) following 72 and 96 hours without LIF. (Representative gel images shown, n=3). (C) FACS plots confirming higher levels of Sox2, Oct3/4, Nanog or Brachyury over 72 and 96 hours following DP-βC or BIO treatment without LIF for 96 hours compared to control cells (Representative images shown, n=2).

To determine how canonical Wnt/β-catenin signaling influences early ES cell differentiation, we activated the pathway either by inhibiting GSK-3 activity using BIO (ATP competitive inhibitor) or by generating stable ES cell lines using the tetracycline regulated system to inducibly express DP-βC with CK1 and GSK-3 phosphorylation sites mutated to prevent proteosomal degradation [43]. Negative regulation was achieved using the Axin stabilizer XAV 939 (XAV). Activation of the pathway in the absence of LIF stimulation (Figure 1A) strongly suppressed differentiation with ES colonies sustaining high levels of alkaline phosphatase and maintaining an undifferentiated phenotype over 72 h (Figure 1B) whereas XAV treatment increased differentiation (Control *45+3.1%*, DP-βC *75+2.1%*, BIO *86+3.2%*, XAV *26+1.5%, n=3*). However canonical Wnt/β-catenin signaling was unable to sustain self-renewal with cells undergoing a degree of differentiation by 96 h of LIF withdrawal. BIO exhibited a greater propensity to suppress differentiation than DP-βC, indicative of GSK-3 ability to modulate other pathways such as the PI3 kinase pathway [44]. To determine early germ-layer fate programs during differentiation we made use of a TaqMan® Mouse Stem Cell Pluripotency Array, which focused on stem cell/pluripotent markers and early differentiation lineage markers. Results indicate that activation of canonical Wnt signaling profoundly alters transcriptional regulation of ES cells during early differentiation induction following LIF removal (Figure 2A). In line with differentiation suppression, genes correlating to ‘stemness’ such as *Nanog, Nr5a2, Oct3/4, Rex1, Tcf* and *Cdx2* were up-regulated by DP-βC expression and GSK-3 inhibition (BIO). Furthermore β-catenin activation strongly induced a mesodermal/mesoendodermal program with characteristic markers *Brachyury* (*T*)*, Pecam 1, Nodal, Myo D1* and *Tcfcp2ll* up-regulated. This was accompanied by a corresponding suppression of endodermal genes (*Col1al, Col2al, Crabp2, Nes* and *Neurod 1*). Semi-quantitative PCR confirmed changes in expression of key genes significantly altered following canonical Wnt/β-catenin signaling. In addition *Sall 1* and *ID 1* genes important for suppressing differentiation were down-regulated following DP-βC expression and BIO treatment. As predicted XAV treatment had the opposite effect, down-regulating *Nanog, Oct3/4, Brachyury, Nodal* and *BMP4* expression (Figure 2B). Flow cytometry analysis of key transcription factors involved is sustaining self-renewal (Sox2, Oct4 and Nanog) in conjunction with Brachyury indicate that in the absence of LIF β-catenin signaling sustains expression of these self-renewal markers whilst substantially up-regulating Brachyury expression (Figure 2C & Figure S1A & S1B). These findings corroborate a role for canonical Wnt signaling in sustaining the ‘stemness’ potential of mesodermal progenitor populations during early embryonic differentiation.

### Active β-catenin indirectly regulates Nanog levels during early differentiation

**Figure 3:**
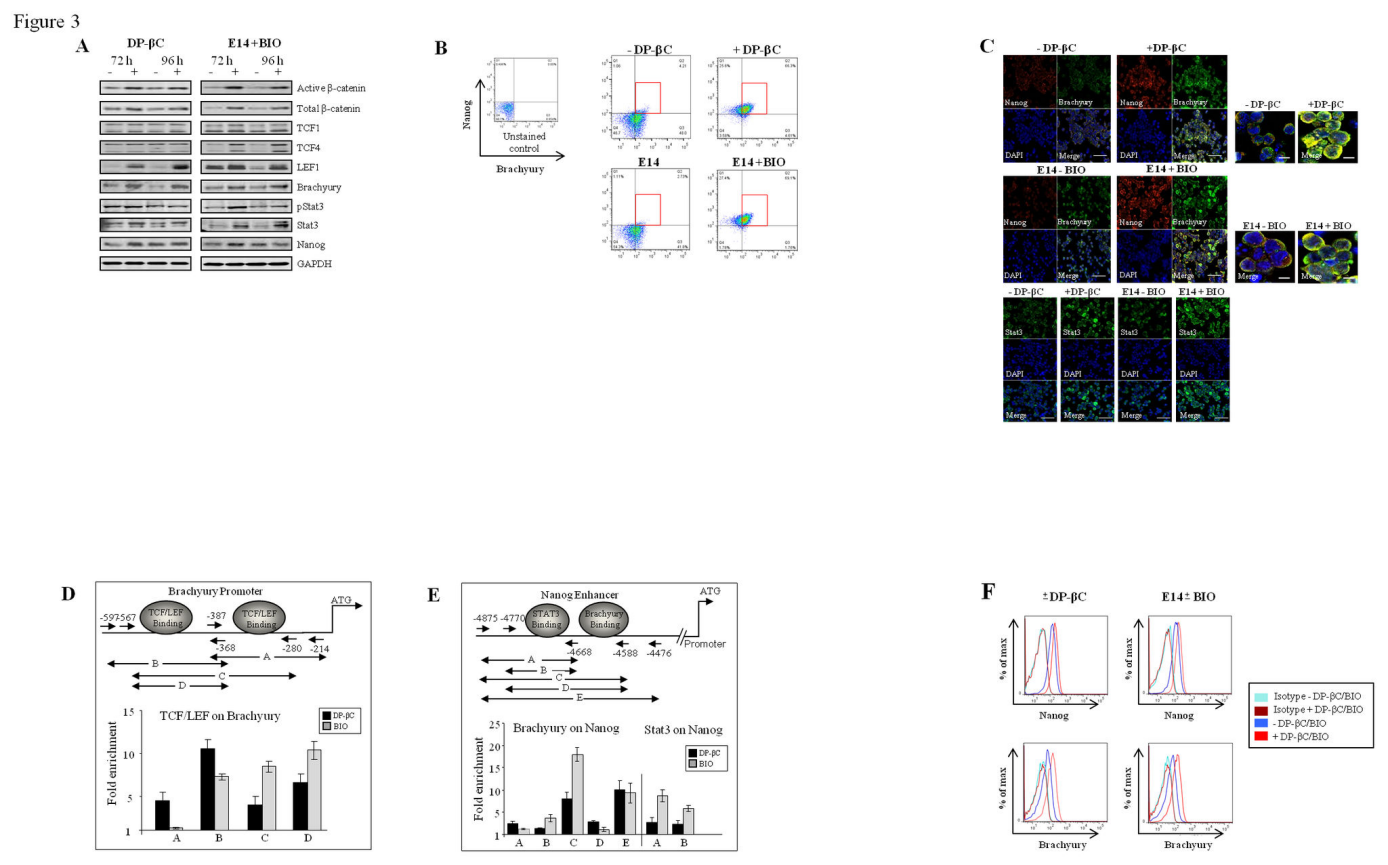
Canonical Wnt signaling biases mesodermal commitment via altered TCF/LEF/Brachyury/Nanog transcription. (A) Protein extracts from DP-βC (+/- Tet) and E14 ES cells +/- BIO) following 96 hour culture in no LIF were immunoblotted to measure levels of active and total β-catenin, TCF/LEF, Brachyury, pStat3, total Stat3 and Nanog with GAPDH used as the loading control. Representative gel images shown, n=3. (B) Flow cytometry showing higher percentage of cells were positive for both Nanog and Brachyury following DP-βC or BIO treatment without LIF for 96 hours compared to control cells (Representative images shown, n=2). (C) Immunofluorescence to show up regulation of self-renewal markers Nanog or Stat3 and mesodermal marker Brachyury following DP-βC expression or BIO treatment. Representative images shown, n=3. (D) Chromatin Immunoprecipitation was performed on DP-βC (+/- Tet) and E14 ES cells (-/+ BIO) cultured in the absence of LIF for 96 hours. The cells were harvested and IP was performed on the sonicated chromatin material using the TCF/LEF antibody sampler kit. The primer sets were designed on regions flanking the TCF/LEF binding sites (Start -597 to Start -368 on the Brachyury promoter). (E) In addition IP was performed using Brachyury or Stat3 antibody on the sonicated chromatin material. The primer sets were designed on regions flanking the Brachyury binding sites (Start -4875 to Start -4476) and Stat 3 binding sites (Start -4875 to Start -4668) on the Nanog enhancer region. Quantitative PCR was performed to measure the relative enrichment (Mean fold enrichment, +/- SEM, n=3). (F) Flow cytometry analysis indicating short-term activation of β-catenin signalling (DP-βC or BIO) rapidly up-regulates Brachyury prior to any significant changes in Nanog levels (Representative images shown, n=2).

To explore the complex interplay involved in stem cell maintenance during differentiation processes we examined the relationship between β-catenin signaling and its ability to regulate TCF/LEF, Brachyury, STAT3 and Nanog levels. Immunoblotting analysis revealed that DP-βC and BIO up-regulated/sustained higher protein levels of TCF/LEF, Brachyury, STAT3 and Nanog during differentiation induction (Figure 3A ). Flow cytometry also indicated that a higher percentage of cells were positive for both Nanog and Brachyury (Figure 3B) with a degree of co-localisation observed between Brachyury and Nanog by immunohistochemistry (Figure 3C). From these data we hypothesised that β-catenin through TCF/LEF1 mediated up-regulation of Brachyury might enhance Nanog levels. Examination of the proximal promoter regions of Brachyury confirmed the two putative TCF/LEF1 consensus binding sites previously reported by Arnold et al. [45], and the predicted Brachyury consensus binding site identified in the Nanog distal enhancer region [46]. ChIP assays were designed to explore these interactions (Table S2). DP-βC expression and BIO treatment resulted in a significant enrichment of the Brachyury proximal promoter from extracts immunoprecipitated with an anti-TCF/LEF antibody cocktail (Figure 3D), indicating canonical Wnt signaling directly regulates Brachyury transcription. In addition significant enrichment of the Nanog distal enhancer was also observed from extracts immunoprecipitated with a specific Brachyury antibody (Figure 3E and Table S3). Similar results were observed following STAT3 immunoprecipitation using primers which span the putative Nanog enhancer STAT3 binding site. Flow cytometry analysis indicates that this is a sequential event with short-term activation of β-catenin signalling rapidly up-regulating brachyury prior to any significant changes in Nanog levels, whereas longer-term activation led to high levels of both brachyury and Nanog (Figure 3F and 2C). Our findings indicate that STAT3 and Brachyury up-regulation through canonical Wnt/β-catenin signaling may potentiate Nanog transcription by converging on its enhancer region.

### β-catenin signaling induces genes involved in primitive hematopoiesis

**Figure 4:**
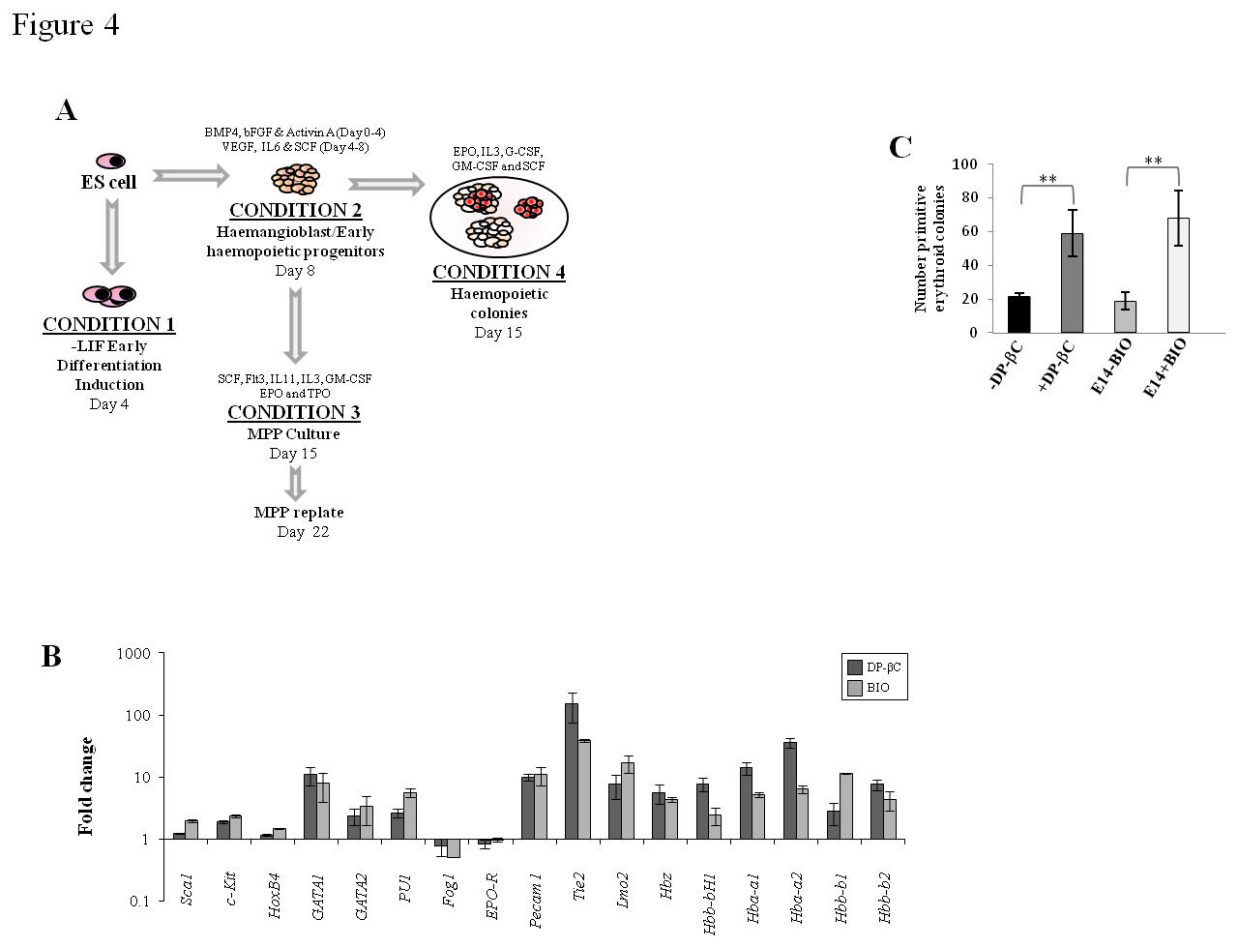
Active β-catenin induces an embryonic erythroid program during early differentiation. (A) Schematic diagram of the different differentiation stages examined. CONDITION 1 early differentiation, CONDITION 2 Hemangioblast/Early hematopoietic progenitors, CONDITION 3 MPP, MEP and GMP differentiation, CONDITION 4 Myeloid colony formation. (B) QRT-PCR demonstrating activation of the canonical Wnt signaling (DP-βC or E14 ES cells + BIO) up-regulates key genes important for establishing early hematopoietic commitment, primitive erythroid specification and embryonic/fetal globin genes during differentiation induction in the absence of LIF (CONDITION 1). Control cells (DP-βC + tet or E14 ES cells -BIO) were used as calibrators and the fold change was calculated using the 2 ^–ΔΔCT^ method (Mean of fold change +/- SEM, n=3). (C) Cells were directed to form EB’s and then cultured in M3434 to promote primitive erythroid colony formation and colonies scored. Active β-catenin signalling (DP-βC or BIO) resulted in a significant increase in primitive erythroid colonies (Mean %, +/- SEM, n=3).

As mesodermal patterning precedes hematopoietic development during embryogenesis, we examined whether active β-catenin induced any master regulators involved in establishing hematopoiesis. Following early differentiation induction by the removal of LIF for 4 days (Figure 4A CONDITION 1), *Sca1, c-Kit* and *HoxB4*, genes involved in HSC formation and self-renewal were up-regulated by activation of the pathway. In addition the specific erythroid lineage marker, *GATA1* and the common lymphoid myeloid progenitor marker, *PU-1* were also up-regulated. Although detected *GATA2, Fog1* and *EpoR* genes involved in erythroid specification were unchanged whereas *SCL* another master regulator of hematopoiesis was undetected (Figure 4B). Early hematopoiesis is accompanied by the production of primitive erythrocytes which express embryonic and fetal hemoglobins. This is a hierarchical system in-part controlled by *LMO2, Tie2* and *Pecam1*. Activation of the Wnt pathway resulted in a significant up-regulation of *LMO2, Tie2, Pecam1* as well as the embryonic and fetal hemoglobin genes (Figure 4B). To explore this further, cells were directed to form EB’s and then cultured in a cytokine cocktail to promote primitive erythroid colony formation. Active β-catenin signaling resulted in a significant increase in primitive erythroid colonies (Figure 4C). These findings indicate that canonical Wnt/β-catenin signal transduction is fundamental for inducing a hematopoietic gene transcription program during early differentiation events in ES cells. 

### β-catenin activation enhances hemangioblast/early hematopoietic progenitor formation

**Figure 5:**
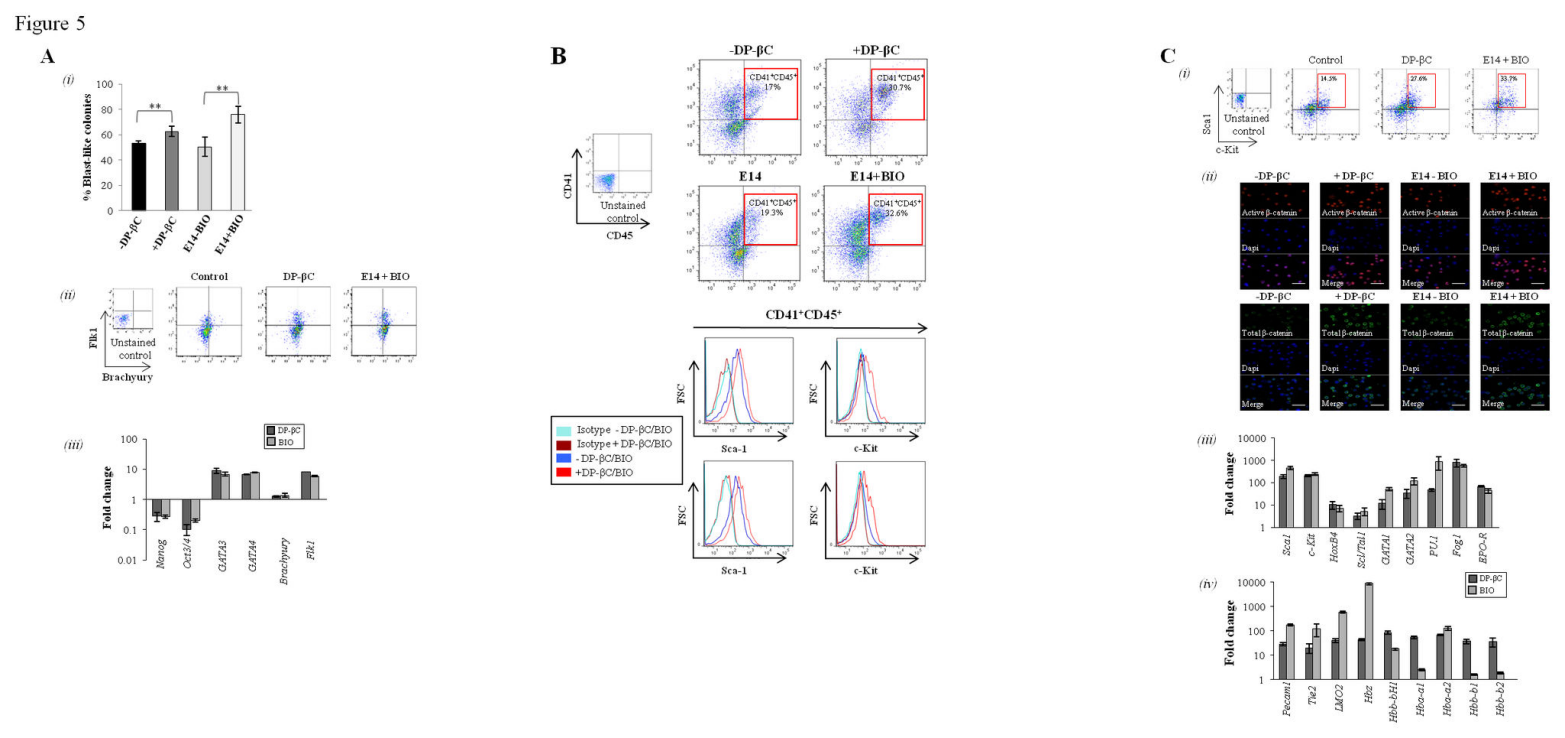
β-catenin activation enhances hemangioblast formation. (A) (i) Scoring of the number of BL-CFC compared to the number of EB’s for each condition by day 8 of differentiation shows activation of the canonical Wnt pathway (DP-βC or BIO) results in higher percentage of BL-CFC compared to control cultures (Mean %, +/- SEM, n=3). (ii) Hemangioblasts/Early hematopoietic progenitors (CONDITION 2) were analysis for expression of mesodermal markers Flk1 and Brachyury by flow cytometry. (iii) RT-PCR analysis of key self-renewal and differentiation genes. (B) Flow cytometry analysis was performed on the hemangioblast/early hematopoietic progenitors to determine the percentage of cells expressing CD41, CD45, c-Kit, Sca1 and Flt3. Gating on the CD41^+^, CD45^+^ population revealed that these early hematopoietic progenitors also expressed the HSC markers Sca1 and c-Kit (Representative images shown, n=3). (C) (i) Total cell population from CONDITION 2 were analyzed for key HSC markers Sca1 and c-Kit by flow cytometry. (ii) The early hematopoietic progenitor populations (Sca1^+^c-Kit^+^ cells) were sorted and Immunofluorescence performed to confirm higher levels of total and active β-catenin in DP-βC –tet and E14 cells + BIO. (iii) RT-PCR analysis of key hematopoietic genes within the Sca-1^+^c-kit^+^ hematopoietic progenitor cells and (iv) erythroid/globin genes. The control cells (DP-βC + tet or E14 ES cells -BIO) were used as calibrators and the fold change was calculated using the 2 ^-ΔΔCT^ method (Mean of fold change +/- SEM, n=3).

Hemangioblasts establish the vasculature and hematopoietic systems during development. To determine how β-catenin activation influences this process, ES cells were directed to undergo hemangioblast/early hematopoietic progenitor formation (Figure 4A CONDITION 2). Previous studies indicate that hemangioblasts are present in blast-like colonies (BL-CFC)[27,31,47], we therefore scored the number of BL-CFC compared to the number of EB’s for each condition. Activation of the canonical Wnt pathway led to a higher percentage of BL-CFC compared to control cultures by day 8 of differentiation (Figure 5A (*i*)). The total cell population generated was analysed for the key endothelial markers Flk1, CD44 and the mesodermal marker brachyury by flow cytometry and revealed that a greater percentage of cells expressed these markers following β-catenin expression or BIO treatment than in the control cultures, indicative of enhanced hemangioblast formation [34] (Figure 5A (*ii*), Figure S2 and summarised in Table 1). Gene transcription analysis also confirmed higher levels of *Brachyury* and *Flk1* expression in line with the increase shown by flow cytometry. In addition *GATA 3 & 4* were up-regulated and *Nanog* and *Oct3/4* down-regulated, confirming enhanced differentiation (Figure 5A (*iii*)). Flow cytometry analysis was also performed to determine the percentage of cells expressing the early hematopoietic progenitor markers CD41, CD45, c-Kit, Sca1 and Flt3. Gating on the CD41^+^, CD45^+^ population revealed that these early hematopoietic progenitors also expressed the HSC markers Sca1 and c-Kit (Figure 5B) with a very high percentage of CD41^+^ cells also co-expressing Sca-1 (Table 1). β-catenin signaling resulted in a significant increase in early hematopoietic/progenitor cells with more cells expressing the blood cell marker CD45 (Figure S2 and summarised in Table 1). 

**Table 1:** FACS analysis: Hemangioblasts.

**% Markers**	**Control**	**DP-βC**	**E14±BIO**
Brachyury	48.7 **^*±*^** 4.4	61.0 **^*±*^** 1.5*	64.7 **^*±*^** 2.3**
Flk1	44.9 **^*±*^** 1.7	63.1 **^*±*^** 4.3*	64.2 **^*±*^** 4.0*
CD44	65.2 **^*±*^** 0.9	72.0 **^*±*^** 2.0*	69.2 **^*±*^** 2.6**
Flt3	49.9 **^*±*^** 1.7	73.0 **^*±*^** 1.4**	72.0 **^*±*^** 2.4**
CD41	46.5 **^*±*^** 10.6	60.5 **^*±*^** 9.2*	62.9 **^*±*^** 7.1*
Sca1	52.6 **^*±*^** 4.2	72.0 **^*±*^** 3.3**	66.1 **^*±*^** 3.2*
c-Kit	52.1 **^*±*^** 3.2	71.3 **^*±*^** 3.1*	69.5 **^*±*^** 3.4*
CD45	25.9**^*±*^** 2.8	39.6 **^*±*^** 2.3**	44.3 **^*±*^** 3.6*
**CD41^±^CD45^±^**	18.2 **^*±*^** 3.2	30.7 **^*±*^** 4.0*	32.6 **^*±*^** 2.7*
Sca1	31.3 **^*±*^** 5.3	48.3 **^*±*^** 3.8*	51.9 **^*±*^** 1.6*
c-Kit	11.1 **^*±*^** 2.0	21.0 **^*±*^** 2.8	16.1 **^*±*^** 2.6
**CD41^±^Sca1^*±*^**	36.5 **^*±*^** 2.5	51.5 **^*±*^** 2.7*	52.7 **^*±*^** 3.3*

Average % ± SEM, n=3 (* p<0.05, ** p<0.005)

To ascertain whether the (Sca1^+^, c-Kit^+^) hematopoietic progenitor populations had alteration in their lineage priming potential following activation of the canonical Wnt pathway we isolated this population by FACs sorting for further analysis (Figure 5C (*i*)). First we confirmed that the activated cells still had higher levels of total and active β-catenin expression (Figure 5C*(ii)*). Next we carried out gene profiling of the Sca1^+^, c-Kit^+^, which revealed β-catenin signaling significantly up-regulated key regulators of HSC formation and self-renewal (*c-Kit, Sca1 & HoxB4*) and master regulators of hematopoietic lineage commitment (*SCL/Tal-1, GATA 1 & 2, PU-1, Fog1 & EpoR*) compared to the control populations (Figure 5C (*iii*)); as well as genes involved in establishing primitive and more definitive erythropoiesis (Figure 5C (*iii & iv*)). A similar gene profile was observed when the total population containing both the hemangioblast/early hematopoietic population was analysed (Figure S3). Overall these data indicate a crucial role for β-catenin in establishing embryonic hematopoiesis. 

### Active β-catenin biases MEP formation

**Figure 6:**
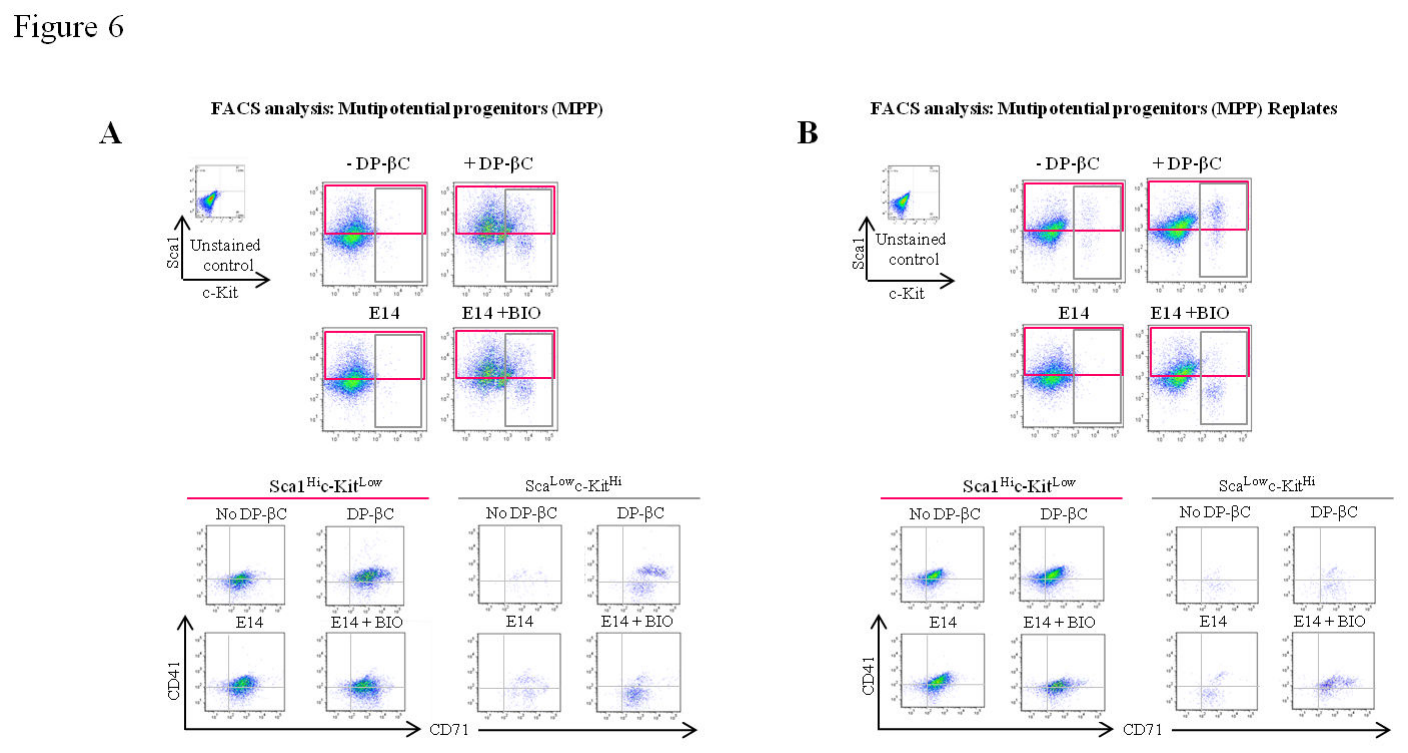
Activation of the canonical Wnt pathway increases MPP and MEP generation. (A) Cells from CONDITION 2 of the differentiation, (DP-βC +/- Tet and E14 ES cells +/- BIO) were cultured in a MPP cocktail for 7 days prior to multi-parameter flow cytometry analysis of total cell population from CONDITION 3. Live cells from CONDITION 3 of the differentiation were gated and the expression of the hematopoietic progenitor markers, Sca1^hi^c-Kit^lo^ and Sca1^lo^c-Kit^hi^ were profiled along with the megakaryocytic marker CD41 and transferrin receptor CD71. (B) MPP population (CONDITION 3) were then replated for an additional 7 days and analyzed for the same panel of markers outlined in (A). Images are representative of 3 independent experiments.

Our early differentiation results indicate that active β-catenin signaling, in the absence of erythropoietin stimulation, primes cells to undergo an erythroid differentiation program. To explore this in more detail we plated the early hematopoietc progenitor cells in a cytokine cocktail which promotes MPP, MEP and GMP formation (Figure 4A, CONDITION 3) [42]. Following seven days in culture, cells were analyzed by multi-parameter flow cytometry for MPP, MEP and GMP populations. The results revealed β-catenin signaling enhanced early hematopoietic progenitor/MPP populations as measured by c-Kit, Sca1 and Flt3 expression (Summarised in Table 2A). In addition high levels of the early erythroid marker CD24 was also observed (Figure S4). In adult mice, levels of Sca1 and c-Kit are routinely used as surrogate markers for HSC/progenitors with long-term and short-term repopulating capacity. We therefore gated on the putative lineage restricted long-term (LT) HSC/progenitor population (Sca1^hi^c-Kit^lo^) in our cultures for CD41 and CD71 expression. Results indicate a significant increase in the percentage of cells expressing these MEP markers in the DP-βC and BIO treated cultures (Figure 6A, middle panel). A similar trend was also observed in the putative short-term (ST) HSC/progenitor population (Sca1^lo^c-Kit^hi^) (Figure 6A, lower panel), indicating β-catenin signaling increases MPP and MEP formation. To assess the self-renewal potential of the MPP populations, cells from the first round were replated. Following a further 7 days in culture, analysis revealed that DP-βC and BIO treatment impeded differentiation with a significantly higher percentage of cells sustaining Sca1 and c-Kit expression (Figure 6B and Table 2B), confirming a higher percentage of cells with LT-HSC/progenitor potential were present in the first round of MPP differentiation. Gating these putative LT-HSC/progenitor and ST-HSC/progenitor populations also revealed a higher percentage of cells co-expressing the MEP markers CD41 and CD71 indicative of the canonical Wnt pathway inducing an erythroid differentiation program (Figure 6B).

**Table 2:** FACS analysis.

**MPP**			
**% Markers**	**Control**	**DP-βC**	**E14±BIO**
Sca1	39.7 **^*±*^** 5.2	54 **^*±*^** 3.6*	57.2 **^*±*^** 3.8**
c-Kit	9.5 **^*±*^** 2	17.4 **^*±*^** 3.1**	18.4 **^*±*^** 2.3*
CD24	54.6 **^*±*^** 5.8	68 **^*±*^** 4.3*	64 **^*±*^** 2.2*
Ter119	1.2 **^*±*^** 0.4	7.3 **^*±*^** 0.3*	4.5 **^*±*^** 0.2*
Gr1	2.9 **^*±*^** 0.9	1.6 **^*±*^** 0.5	1.1 **^*±*^** 0.4
Flt3	0.9 **^*±*^** 0.2	2.7 **^*±*^** 0.3	2.2 **^*±*^** 0.7
**Sca1^hi^Kit^lo^**			
CD45	66.0 **^*±*^** 7.9	78.7 **^*±*^** 5*	85 **^*±*^** 1**
CD41	39.4 **^*±*^** 3.1	60.2 **^*±*^** 3.7**	57.3 **^*±*^** 3*
CD71	58.1 **^*±*^** 3.4	71.3 **^*±*^** 2.5**	68.9 **^*±*^** 4.3*
**Sca1^lo^Kit^hi^**			
CD45	14.4 **^*±*^** 2.1	20.6 **^*±*^** 2.3	25.1 **^*±*^** 1.6*
CD41	27.2 **^*±*^** 2.7	43 **^*±*^** 4.5*	41.3 **^*±*^** 2.1*
CD71	60.9 **^*±*^** 6.1	73.7 **^*±*^** 3.9**	77 **^*±*^** 3.6*
**MPP Replates**			
**% Markers**	**Control**	**DP-βC**	**E14±BIO**
Sca1	44.6 **^*±*^** 3	55.6 **^*±*^** 3.4*	57.3 **^*±*^** 4*
c-Kit	7.6 **^*±*^** 2.5	13.9 **^*±*^** 2.2*	17.4 **^*±*^** 3*
CD24	65.7 **^*±*^** 6.3	74.3 **^*±*^** 3.8*	71.4 **^*±*^** 4.5**
Ter119	0.2 **^*±*^** 0.0	0.04 **^*±*^** 0.0	1.2 **^*±*^** 0.6
Gr1	4.2 **^*±*^** 0.3	0.7 **^*±*^** 0.2*	2.1 **^*±*^** 0.6*
Flt3	0.3 **^*±*^** 0.1	0.03 **^*±*^** 0.0	1.3 **^*±*^** 0.1
**Sca1^hi^Kit^lo^**			
CD45	82.6 **^*±*^** 3.5	92.5 **^*±*^** 3.2*	88.7 **^*±*^** 4.2*
CD41	49.1 **^*±*^** 2.4	59 **^*±*^** 3.5	63.6 **^*±*^** 3.4*
CD71	60.2 **^*±*^** 4.5	64.3 **^*±*^** 5.6*	68.4 **^*±*^** 3.7*
**Sca1^lo^Kit^hi^**			
CD45	21.0 **^*±*^** 2.3	13.1 **^*±*^** 2.2*	15.2 **^*±*^** 2.4*
CD41	30.3 **^*±*^** 2.7	43.0 **^*±*^** 3.6**	38.9 **^*±*^** 4*
CD71	62.5 **^*±*^** 3.7	68.3 **^*±*^** 4.8*	70.4 **^*±*^** 3.3

Average % ± SEM, n=3 (* p<0.05, ** p<0.005)

### Active β-catenin permits granulocyte-macrophage colony formation

**Figure 7:**
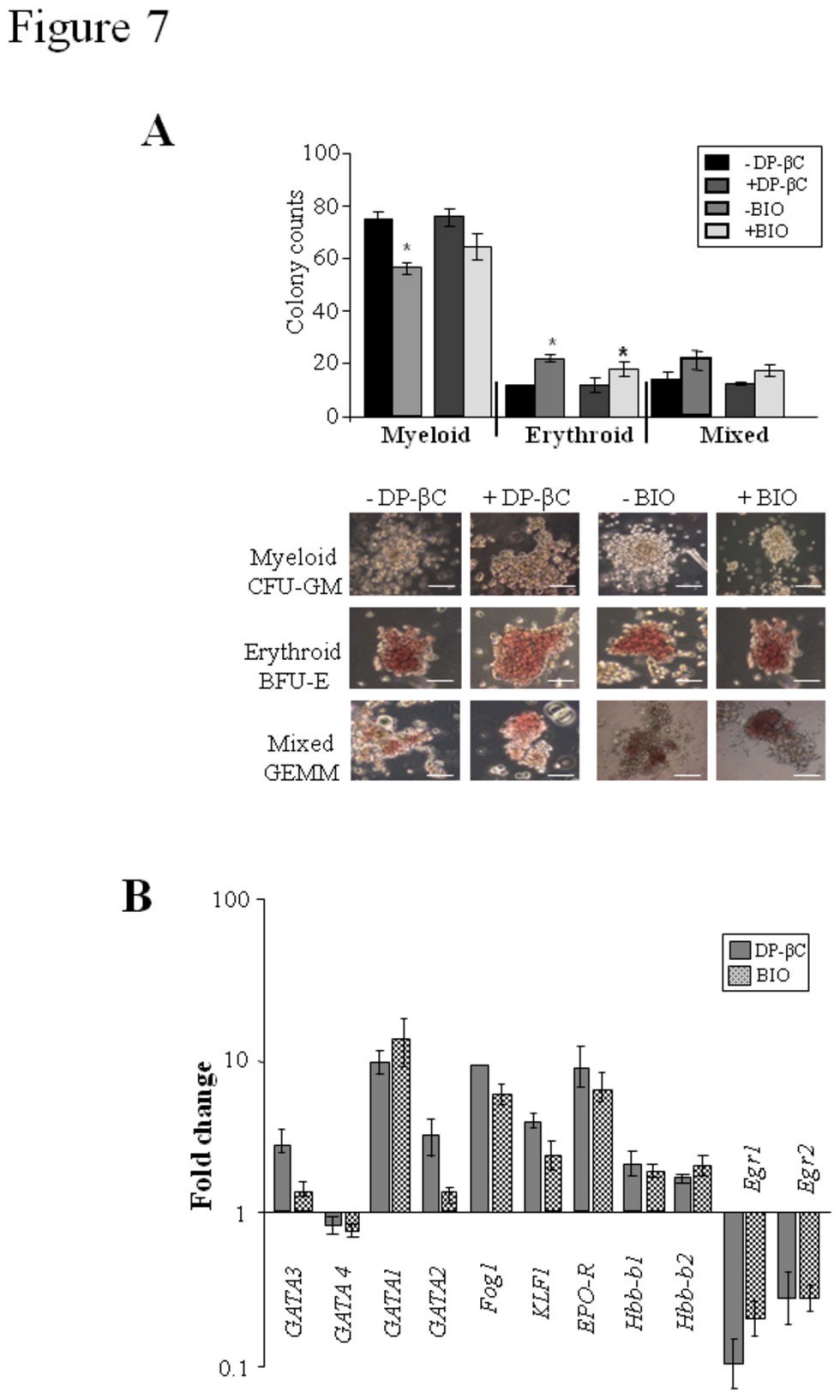
β-catenin permits granulocytic/macrophage colony formation. (A) Cells from CONDITION 2 of the differentiation, were directed to undergo myeloid colony formation and the number of GM-CFU, GEMM, and BFU-E colonies counted-CONDITION 4. Graph represents the Mean % +/- SEM, n=3. Representative pictures of hematopoietic colonies x 10 magnification, scale bar 100 µM. (B) Gene expression profiling of erythroid and myeloid genes. The control cells (DP-βC +Tet or E14 ES cells -BIO) were used as calibrators and the fold change was calculated using the 2 ^–ΔΔCT^ method (Mean of fold change +/- SEM, n=3).

To determine whether active β-catenin blocked granulocyte-macrophage (GM) colony formation we employed a cytokine cocktail which strongly promotes CFU-GM whilst permitting BFU-E and GEMM formation (Figure 4A CONDITION 4). As predicted by the strong erythroid program induced during differentiation induction by both DP-βC and BIO, a significant enhancement in BFU-E and GEMM formation was observed (Figure 7A). However, high numbers of CFU-GM still formed in both the DP-βC and BIO cultures. These changes were supported by an up-regulation of the HSC regulators *GATA1, Klf-1*, *Fog1* and *EPO-R* along with the adult globin genes *Hbb-b1, Hbb-b2*, whereas the monocytic specific genes *Egr1* & *2* (Figures 7B) were down-regulated.

## Discussion

The canonical Wnt/GSK3β/β-catenin pathway is known to act synergistically with the LIF/STAT-3 pathway to improve ES cell self-renewal [48-50]. However, information is limited on its role during early differentiation decisions, especially embryonic hematopoiesis. Our data indicates that during differentiation induction following LIF withdrawal, β-catenin suppresses this process whilst promoting mesodermal cell fate determination. Cells treated with BIO or induced to express active β-catenin, retained alkaline phosphatase staining and had higher expression levels ofkey pluripotency markers Oct3/4, Nanog and Sox2. These findings were confirmed at the transcription level with pluripotency genes, especially *Oct3/4*, *Nanog*, *Sox2* and *Rex1*all up-regulated. Genes involved in mesodermal patterning were also up-regulated following activation of the β-catenin pathway. In particular the T-box targets *Eomesogermin* (*Eomes*) and *Braychury* (*T*) were significantly up-regulated. During mouse development *Eomes* is the earliest regulator of mesoderm formation being required for both embryonic and extraembryonic mesoderm formation [51]. *T* overlaps with *Eomes*, with *T* also implicated in later processes such as left/right axis determination and somite segmentation [52]. In addition mesoendoderm specification genes were also up-regulated, these included; *Fgf4* expressed in intermediate mesoderm [53], *GATA* 4 and 6 expressed in lateral mesoderm during organogenesis [54,55], *Lefty 1* & *2* and *Nodal* involved in left/right axis determination, and lateral plate mesoderm formation during gastrulation [56,57]. A previous study using β-catenin knock-out ES cells showed an inverse correlation to our findings with genes including *Nanog*, *Rex1, Lefty1* and *Lefty2* all down-regulated [57]. These data support the idea that β-catenin is intricately involved in regulating the expression of *Nanog*. Using flow cytometry and ChIP technology we demonstrate that a complex relationship exists between β-catenin signaling and LEF/TCF/Brachyury/Nanog levels. Following β-catenin activation, TCF/LEF could potentially directly bind to its consensus sites in the Brachyury promoter [45] leading to the higher levels of Brachyury expression we observe. This in turn may enable Brachyury to bindto the distal enhancer site of Nanog downstream of STAT3, to potentiate Nanog expression. Our data demonstrates that Brachyury up-regulation precedes Nanog supporting this potential mechanism. These findings are also supported by a previous study whereby BMP in combination with LIF signaling was able to indirectly augment Nanog expression through Brachyury, causing ES cells to become early-mesoderm cells [46,58]. Our findings provide a potential mechanism through which active β-catenin sustains stem cell potential during early mesoderm specification. These findings contribute to our understanding of how Wnt signalling controls ES fate decisions. Previous studies indicate that Wnt in combination with LIF signalling is fundamental for sustaining pluripotency and germ line transmission. When Wnt signalling is blocked, LIF in the absence of Wnt signalling results in ES cells differentiating into epiblast stem cells [59] whereas our findings indicate that Wnt signaling in the absence of LIF results in the induction of a mesodermal cell fate.

Recent studies report Wnt, along with Activin and BMP signals are important for hematopoietic development, with inhibition of Wnt signaling preventing the formation of Flk1^+^ mesoderm followed by a reduction in CD41^+^ primitive erythroid colony formation [35]. Conversely Wnt pathway activation enhanced hematopoiesis through up-regulation of *SCL* and *T3 globin* transcription [9]. Wnt family members are highly expressed in the YS, AGM and FL during embryogenesis with several members including non-canonical Wnt 4, 5a and 16 and canonical Wnt 3a known to be important for establishing embryonic hematopoiesis [1,5,7-9,13,26,60]. Indeed Wnt signaling is essential for HSC generation from the AGM region at E10.5 [14], however how canonical Wnt signaling specifies early hematopoietic commitment is still unclear. We therefore used our ES differentiation system and performed detailed gene analysis of the transcriptional complexes involved in establishing primitive hematopoiesis in order to understand how the Wnt pathway regulates this process early on during differentiation. In cells induced to differentiation by the removal of LIF for 4 days we clearly demonstrate that β-catenin signaling up-regulates *Kit, CD34* and *Pecam 1*, genes which mark the onset of primitive hematopoietic cell establishment from extraembryonic mesoderm and YS development [34,61]. Along with significantly up-regulating key genes involved in establishing primitive erythropoiesis including; *GATA1, Pecam 1, Tie2* and *LMO2* with no change in *GATA2, Fog1* and *EpoR* levels. It is known that primitive erythroid cells arise from *GATA1* and *2* expressing cells in the YS which also have endothelial potential and express the endothelial markers *Flk1, Tie2* and *Pecam 1* [61]. *GATA2* null mice are embryonic lethal, due to severe anemia during the early phase of YS hematopoiesis (E10-11) [62]. Expression of *GATA2* precedes *GATA1* and must decrease as *GATA1* expression increases to enable erythroid differentiation. Another transcription factor essential for primitive hematopoiesis and induced by GATA2 is LMO2. *LMO2* null mice die around E9 of severe anemia, with a lack of any YS hematopoiesis [63,64]. TCF transcription factors in concert with cell specific master regulators, including GATA 1 and 2 and C/EBPα, have recently been shown to selectively bind to the enhancer region of lineage-distinctive genes to promote erythroid and myeloid differentiation programs. Following Wnt signalling TCF7L2 co-operated with GATA2 to increase LMO2 transcription [65]. LMO2 then acts in a complex with *GATA1* and *SCL* to facilitate DNA binding and erythroid gene transcription [66]. Key erythroid genes regulated by GATA1 and 2 include the globins, *EKLF, Fog*, *EpoR* and heme biosynthesis enzymes [67,68]. Considering the essential role these transcription factors play in generating primitive erythroid cells, we also examined globin gene expression. The initially expressed globin genes are *Hbz* and *Hbb-βH1* which are then superseded by the *Hba-α1*, *Hba-α2* and *ɛy*- globin genes, as proerythroblasts at E7.5 transition to reticulocytes at E15.5 [69]. Our data established a sequential up-regulation of embryonic and fetal globin genes by Wnt signaling during early hematopoietic specification, with the adult globins being up-regulated at the later stages of hematopoietic differentiation. This is the first report of β-catenin signaling affecting globin switching and corroborate the Nostro et al. demonstrated that over expression of β-catenin enhanced primitive erythroid progenitor formation [35]. Taken together these findings validate a role for this pathway in orchestrating primitive erythropoiesis.

The transition from primitive to more definitive hematopoiesis involves the formation of hematopoietic progenitors with self-renewing potential. In order to explore how β-catenin influenced this process we directed ES cells to form hemangioblast/early hematopoietic progenitors. Active β-catenin significantly augmented this process with 60-75% of the cells expressing the early HSC/progenitor markers CD41, c-Kit, Sca1 and Flt3 compared to ~50% in the control cultures, with 40% of the cells also expressing the pan-hematopoietic marker CD45 compared to 25% in the control cultures. Isolation of the Sca1^+^c-Kit^+^ population followed by gene profiling revealed that transcription factors involved in HSC specification and self-renewal were strongly up-regulated in these early hematopoietic progenitors by β-catenin signaling, including *SCL/Tal1*, essential for initiating the hematopoietic program at this stage [70]. Genes essential for erythroid lineage priming were also up-regulated including the adult globin genes necessary to switch from primitive to definitive erythropoiesis. Given that during CONDITION 1 of differentiation the cells had been cultured in the absence of growth factors and by CONDITION 2 in growth factors which promote mesoderm followed by hemagioblast/early hematopoietic progenitor formation in the absence of erythropoietin, these findings provide important insight into the role canonical Wnt signaling plays in establishing the hematopoietic system during early development.

In order to determine how influential canonical Wnt signaling was in predisposing cells to undergo erythropoiesis, the early hematopoietic progenitor population were cultured in a cytokine cocktail to promote MPP formation [42]. Active β-catenin resulted in a higher percentage of cells sustaining expression of the early HSC/progenitor markers Sca1/c-Kit, along with the immature hematopoietic lineage marker CD24, which persists during erythrocyte differentiation [71]. CD41 a Runx-1/SCL regulated marker [72–75] was also highly expressed following β-catenin signaling. In combination with CD71, CD41 expression can be used to characterise MEP progenitors. Following MPP culture, active β-catenin increased the number of cells with MEP potential (Ter119^lo^CD41^hi^CD71^hi^) within both the Sca1^hi^c-Kit^lo^ and Sca1^lo^c-Kit^hi^ populations. Following MPP replating, β-catenin signaling sustained the number of Sca1^hi^c-Kit^lo^ cells. This is in accordance with the mouse models where conditional expression of active β-catenin in the hematopoietic system results in a large expansion of Sca1^+^c-Kit^+^ LT-HSC. Analysis revealed that these HSCs were unable to sustain long-term reconstitution ability following transplantation. This was due to β-catenin pushing cells into cycle increasing proliferation and leading to the accumulation of undifferentiated progenitors which eventually resulted in HSC exhaustion. In addition the progenitors generated exhibited defective GM colony forming ability and enhanced potential to undergo erythroid differentiation *in vitro* and *in vivo* reflected by higher erythroid cell numbers in the bone marrow and spleen [21,22]. Recent evidence indicates that the level of canonical Wnt signaling can greatly affect HSC behaviour, with moderate to low levels of activation being advantageous whereas sustained high levels are detrimental leading to a differentiation block and an inability of HSC to reconstitute lethally irradiated recipients [26]. In our *in vitro* model cytokine signaling through G-CSF and GM-CSF was able to partially overcome this block in GM colony formation, supporting the idea that although β-catenin promotes an erythroid transcriptional program, the HSC/progenitor populations still retain the capacity to undergo granulocyte/macrophage differentiation. 

Overall we demonstrate the transcriptional changes canonical Wnt signaling alters to orchestrate early developmental hematopoietic stages. Our data clearly defines a role for β-catenin in directing ES cells to form mesodermal progenitors and enhancing the formation of hematopoietic progenitors with erythrocytic potential. In addition β-catenin induced expression of genes involved in maintaining stem cell potential during these early differentiation decisions, an essential process necessary for sustaining the rapid proliferation required during the establishment of the hematopoietic system. 

## Supporting Information

Figure S1 **Wnt signaling up-regulates brachyury levels in the absence of LIF** (**A**). Dot plots and graphs showing DP-βC expression or BIO treatment increases the percentage of cells expressing Brachyury over 72 and 96 hours following LIF withdrawal. (Representative images shown, graph of Mean % +/- SEMs, n=3). *Activating* *the β-catenin* *pathway* *sustains* *Nanog, and* *Oct3/4* *expression* *following* *LIF* removal (**B**). Representative histograms showing DP-βC expression or BIO treatment sustains the percentage of cells expressing Nanog and Oct3/4 over 72 and 96 hours to similar levels as observed at 20h following LIF withdrawal.
(TIF)

Figure S2 **β-catenin activation enhances hemangioblast and hematopioetic markers during differentiation.** FACS analysis to measure percentage of cells expressing hemangioblast and hematopoietic markers following DP-βC expression or BIO treatment. Activation of the pathway up-regulates the mesodermal marker Brachyury along with mesoendodermal Flk1 and CD44. Analysis also shows that HSC markers (Sca1 & c-Kit), myeloid progenitor marker (Flt3) and hematopoietic cell marker (CD45) are all up-regulated by activation of the canonical Wnt pathway (Representative histograms shown, n=3).
(TIF)

Figure S3 **Early hematopoietic progenitors up-regulate early and late erythroid genes following canonical signalling.** RT-PCR analysis for the total hemangioblast/hematopoietic progenitor populations (*i*) key hematopoietic genes (ii) erythroid/globin genes. The control cells (DP-βC + tet or E14 ES cells -BIO) were used as calibrators and the fold change was calculated using the 2 ^-ΔΔCT^ method (Mean of fold change +/- SEM, n=3).
(TIF)

Figure S4 **Activation of the canonical Wnt pathway increases MPP and MEP generation.** Multi-parameter flow cytometry analysis of the MPP populations following DP-βC expression and BIO treatment. The dot plots represent the expression profiles for CD24, Ter119, Gr1 and Flt3 after the first round of MPP formation and following replating for an additional 7 days in the MPP cytokine cocktail. Representative dot plots shown, n=3.
(TIF)

Table S1 **Table showing the CT, ΔΔCT and Fold change values for ChIP data.** Gels depicting levels of Brachyury or Nanog following IP for the TCF or the Brachyury promoter respectively between treated (DP-βC or BIO) and control samples with Rabbit IgG used as a negative control.
(TIF)

Table S2 **Table showing the forward and the reverse primers used in the ChIP assays in this study and the sizes for each product with respect to the start site.**
(TIF)

Table S3 **Tables showing the primer sequence, accession numbers and the cycling conditions for the forward and reverse primers used in this study.**
(TIF)
