# Supplementary figures and images for: Canonical Wnt Signaling Promotes Early Hematopoietic Progenitor Formation and Erythroid Specification during Embryonic Stem Cell Differentiation
Source: PLoS One. 2013 Nov 26;8(11):e81030. doi: 10.1371/journal.pone.0081030 (PMC3850021; doi:10.1371/journal.pone.0081030)

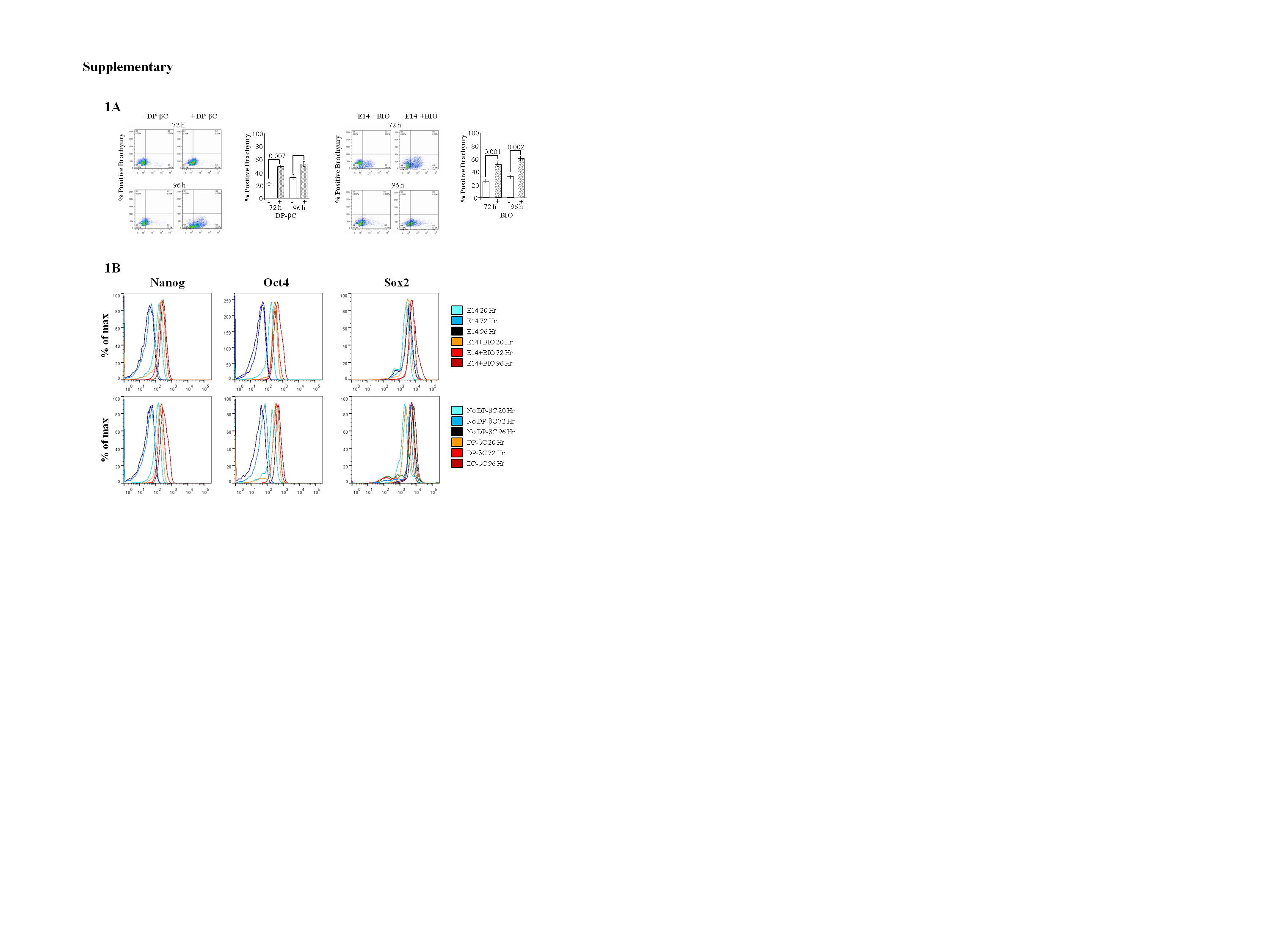

Supplement: Figure S1 — Wnt signaling up-regulates brachyury levels in the absence of LIF (A). Dot plots and graphs showing DP-βC expression or BIO treatment increases the percentage of cells expressing Brachyury over 72 and 96 hours following LIF withdrawal. (Representative images shown, graph of Mean % +/- SEMs, n=3). Activating the β-catenin pathway sustains Nanog, and Oct3/4 expression following LIF removal (B). Representative histograms showing DP-βC expression or BIO treatment sustains the percentage of cells expressing Nanog and Oct3/4 over 72 and 96 hours to similar levels as observed at 20h following LIF withdrawal. (TIF) [file pone.0081030.s001.tif]

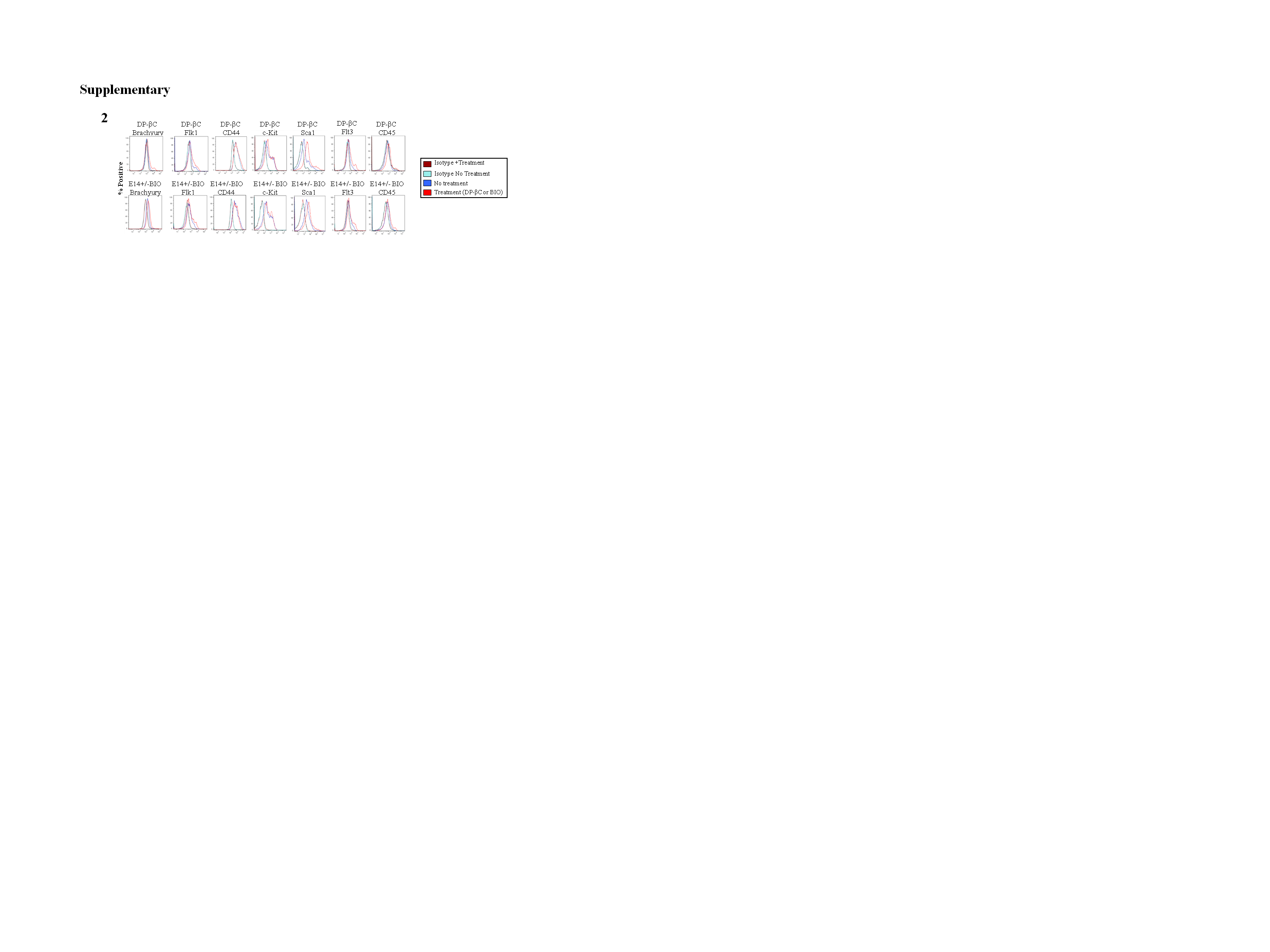

Supplement: Figure S2 — β-catenin activation enhances hemangioblast and hematopioetic markers during differentiation. FACS analysis to measure percentage of cells expressing hemangioblast and hematopoietic markers following DP-βC expression or BIO treatment. Activation of the pathway up-regulates the mesodermal marker Brachyury along with mesoendodermal Flk1 and CD44. Analysis also shows that HSC markers (Sca1 & c-Kit), myeloid progenitor marker (Flt3) and hematopoietic cell marker (CD45) are all up-regulated by activation of the canonical Wnt pathway (Representative histograms shown, n=3). (TIF) [file pone.0081030.s002.tif]

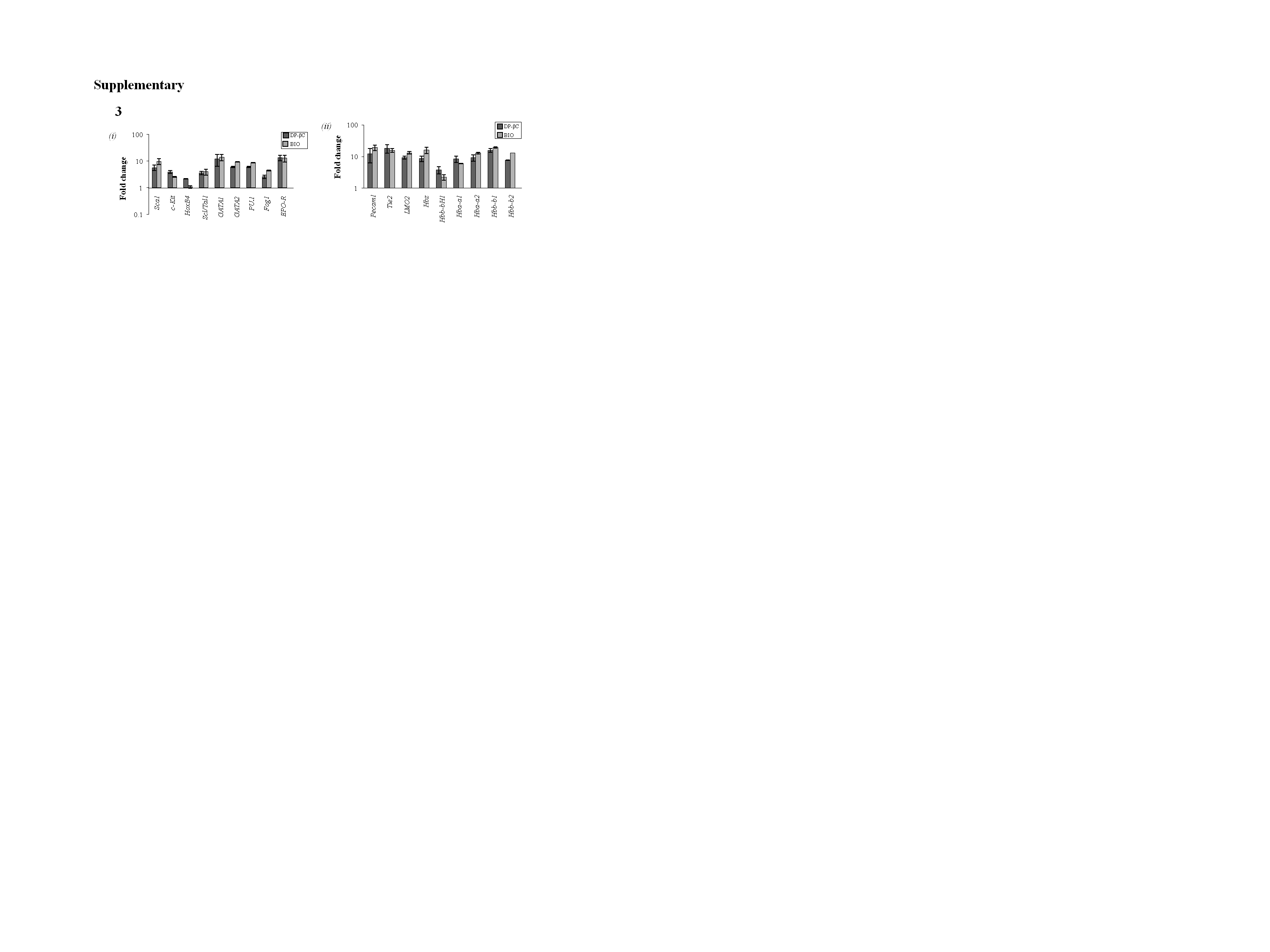

Supplement: Figure S3 — Early hematopoietic progenitors up-regulate early and late erythroid genes following canonical signalling. RT-PCR analysis for the total hemangioblast/hematopoietic progenitor populations (i) key hematopoietic genes (ii) erythroid/globin genes. The control cells (DP-βC + tet or E14 ES cells -BIO) were used as calibrators and the fold change was calculated using the 2 -ΔΔCT method (Mean of fold change +/- SEM, n=3). (TIF) [file pone.0081030.s003.tif]

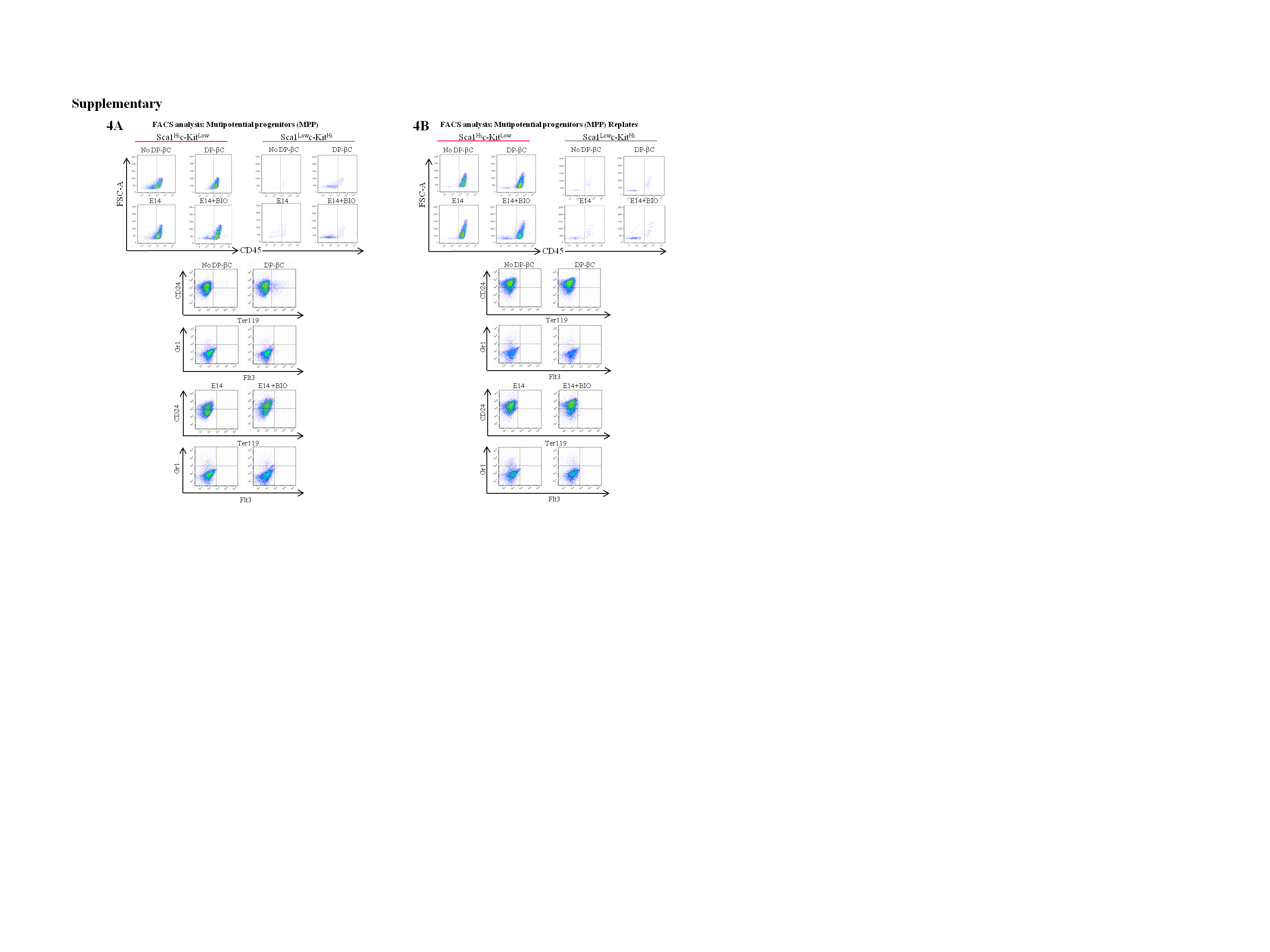

Supplement: Figure S4 — Activation of the canonical Wnt pathway increases MPP and MEP generation. Multi-parameter flow cytometry analysis of the MPP populations following DP-βC expression and BIO treatment. The dot plots represent the expression profiles for CD24, Ter119, Gr1 and Flt3 after the first round of MPP formation and following replating for an additional 7 days in the MPP cytokine cocktail. Representative dot plots shown, n=3. (TIF) [file pone.0081030.s004.tif]

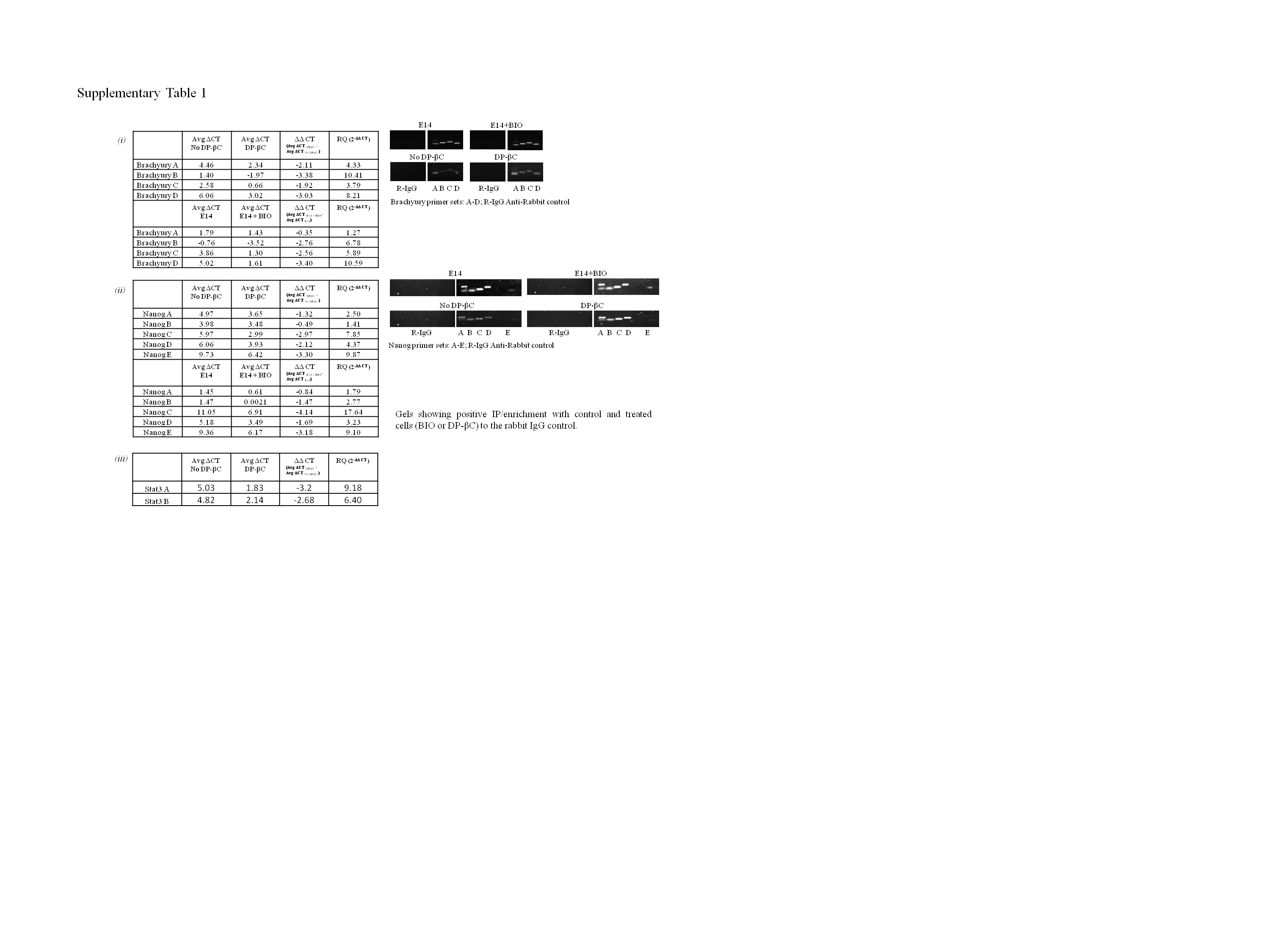

Supplement: Table S1 — Table showing the CT, ΔΔCT and Fold change values for ChIP data. Gels depicting levels of Brachyury or Nanog following IP for the TCF or the Brachyury promoter respectively between treated (DP-βC or BIO) and control samples with Rabbit IgG used as a negative control. (TIF) [file pone.0081030.s005.tif]

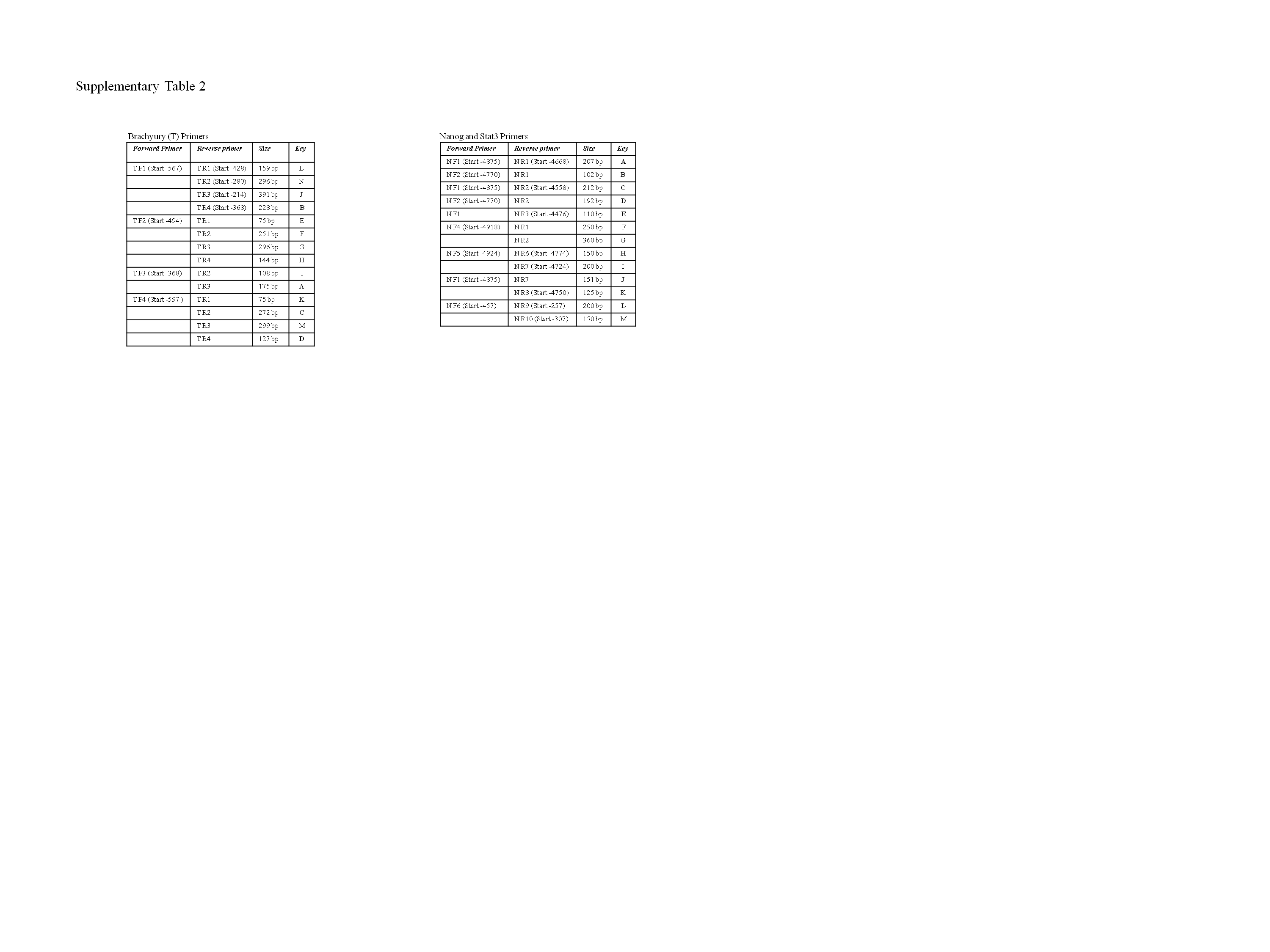

Supplement: Table S2 — Table showing the forward and the reverse primers used in the ChIP assays in this study and the sizes for each product with respect to the start site. (TIF) [file pone.0081030.s006.tif]

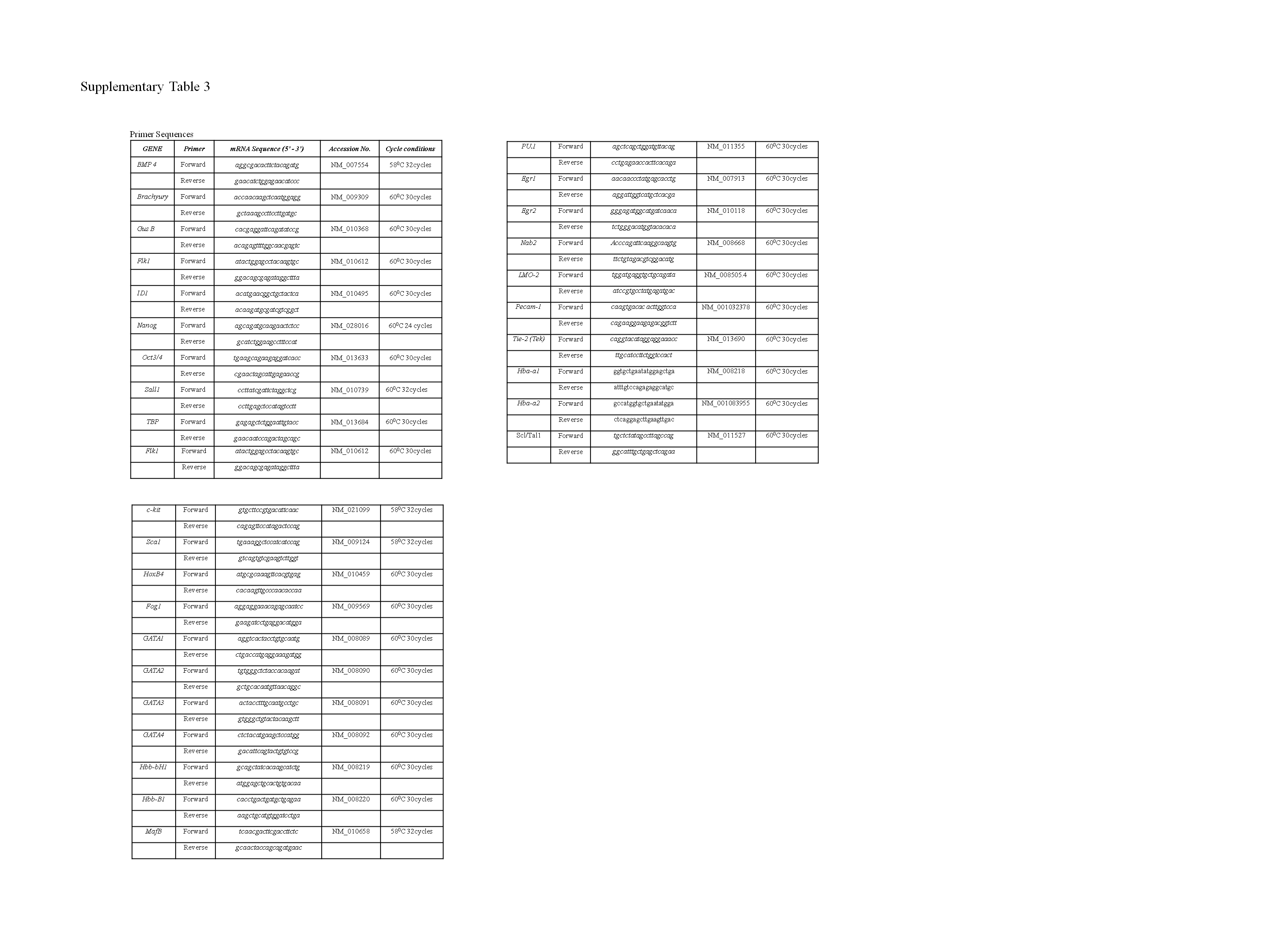

Supplement: Table S3 — Tables showing the primer sequence, accession numbers and the cycling conditions for the forward and reverse primers used in this study. (TIF) [file pone.0081030.s007.tif]
